# Supplementary material for: Improved kala-azar case management through implementation of health facility-based sentinel sites surveillance system in Bihar, India
Source: PLoS Negl Trop Dis. 2021 Aug 24;15(8):e0009598. doi: 10.1371/journal.pntd.0009598 (PMC8384155; doi:10.1371/journal.pntd.0009598)
Supplement: Attachment — Submitted filename: PNTD-D-20-01759_reviewer1.pdf [file pntd.0009598.s001.pdf]

PLOS Neglected Tropical Diseases

Improved Kala-azar Case Management through Implementation of Health Facility-Based Sentinel Sites Surveillance System in Bihar, India

--Manuscript Draft--

**Manuscript Number:**	PNTD-D-20-01759
**Full Title:**	Improved Kala-azar Case Management through Implementation of Health Facility-Based Sentinel Sites Surveillance System in Bihar, India
**Short Title:**	Kala-azar Case Management through Health Facility-Based Sentinel Sites Surveillance System
**Article Type:**	Research Article
**Keywords:**	Kala-azar; Visceral leishmaniasis; Sentinel site surveillance; Post kala-azar dermal leishmaniasis; Co-infection; Hotspot; Kala-azar elimination; Case management.

Abstract:

Background

Visceral leishmaniasis (VL), also known as kala-azar, is a neglected vector-borne disease, targeted for elimination, but several affected blocks of Bihar are posing challenge with the high incidence of cases, and moreover the disease is spreading in newer areas. On the other side, no accurate data on the burden of Post Kala-azar Dermal Leishmaniasis (PKDL) and co-infections are available under current surveillance system, which might emerge as a serious concern. Heath facility-based sentinel site surveillance system was proposed to do a proper accounting of VL, PKDL and co-infection morbidity, mortality, diagnosis, case management, hotspot identification and monitoring the impact of elimination interventions.

Methodology/Principal Findings

Sentinel site kala-azar surveillance was established and activated at thirteen health facilities in Bihar, using stratified sampling technique during 2011 to 2014. Data were collected through specially designed proforma from all patients attending the outpatient departments of sentinel sites. Among 20968 symptomatic cases attended sentinel sites, 2996 cases of KA and 53 cases of PKDL were registered from 889 endemic villages. The proportion of new and old cases was 86.1% and 13.9% respectively. A statistically significant difference was observed for reduction in KA incidence from 4.13/10000 in 2011 to 1.75/10000 in 2014 ($p<0.001$). There were significant increase (0.08, 0.10 per 10 000 population) in the incidences of PKDL and co-infection respectively in the year 2014 as compared to that of 2011 (0.03, 0.06 per 10 000 population). The proportion of HIV-VL co-infection was significantly higher (1.6%; $p<0.05$) as compared to other co-infections. Proportions of male in all age groups were higher and found statistically significant (Chi-square test = 7.6; $P=0.026$).Utilization of laboratory services was greatly improved. Friedman test showed statistically significant difference between sensitivity of different anti kala-azar drugs ($F = 25.0$, $P=0.004$).The initial and final cure rate of AmBisome was found excellent (100%). The results of the signed rank sum test showed significant symmetry of unresponsiveness rate ($P=0.03$). Similarly, relapse rate of SAG was also found significantly higher as compared to other drugs (95%CI 0.2165 to 19.7035; $P=0.03$). A statistically significant difference was found ($p<0.001$) between villages having 1-2 cases (74%) and villages with 3-5 cases (15%). Significantly higher proportion (95%) of cases were captured by Govt. surveillance (HMIS) ($p<0.001$), as compared to private providers (5%).

Conclusions/Significance

Establishment of a sentinel site based kala-azar surveillance system in Bihar, India effectively detected the rising trend of PKDL and co-infections and captured complete and accurate patient level data. Further, this system may provide a model for improving laboratory services and KA, PKDL and co-infection case management in other health facilities in Bihar without further referral. Program Managers may use these results for evaluating program’s effectiveness and improving program management. It may provide an example for changing the practices of health care workers in Bihar and set a bench mark of high quality surveillance data in a resource limited setting. However, generalizability of this sentinel surveillance finding to other context remains a major limitation of this study.

Powered by Editorial Manager® and ProduXion Manager® from Aries Systems Corporation

Additional Information:	
**Question**	**Response**
**Financial Disclosure**<br><br>Enter a financial disclosure statement that describes the sources of funding for the work included in this submission. Review the submission guidelines for detailed requirements. View published research articles from *PLOS NTDs* for specific examples.<br><br>This statement is required for submission and **will appear in the published article** if the submission is accepted. Please make sure it is accurate.<br><br>**Unfunded studies**<br><br>Enter: *The author(s) received no specific funding for this work.*<br><br>**Funded studies**<br><br>Enter a statement with the following details:<br>• Initials of the authors who received each award<br>• Grant numbers awarded to each author<br>• The full name of each funder<br>• URL of each funder website<br>• Did the sponsors or funders play any role in the study design, data collection and analysis, decision to publish, or preparation of the manuscript?<br>• **NO** - Include this sentence at the end of your statement: *The funders had no role in study design, data collection and analysis, decision to publish, or preparation of the manuscript.*<br>• **YES** - Specify the role(s) played.<br><br>* typeset	The funders had no role in study design, data collection and analysis, decision to publish, or preparation of the manuscript.
**Competing Interests**	The authors have declared that no competing interests exist.

Use the instructions below to enter a competing interest statement for this submission. On behalf of all authors, disclose any competing interests that could be perceived to bias this work—acknowledging all financial support and any other relevant financial or non-financial competing interests.<br><br>This statement **will appear in the published article** if the submission is accepted. Please make sure it is accurate. View published research articles from *PLOS NTDs* for specific examples.<br><br>**NO authors have competing interests**<br><br>Enter: *The authors have declared that no competing interests exist.*<br><br>**Authors with competing interests**<br><br>Enter competing interest details beginning with this statement:<br><br>*I have read the journal's policy and the authors of this manuscript have the following competing interests: [insert competing interests here]*<br><br>* typeset	
**Data Availability**<br><br>Authors are required to make all data underlying the findings described fully available, without restriction, and from the	Yes - all data are fully available without restriction

time of publication. PLOS allows rare exceptions to address legal and ethical concerns. See the PLOS Data Policy and FAQ for detailed information.<br><br>A Data Availability Statement describing where the data can be found is required at submission. Your answers to this question constitute the Data Availability Statement and **will be published in the article**, if accepted.<br><br>**Important:** Stating 'data available on request from the author' is not sufficient. If your data are only available upon request, select 'No' for the first question and explain your exceptional situation in the text box.<br><br>Do the authors confirm that all data underlying the findings described in their manuscript are fully available without restriction?	
**Describe where the data may be found in full sentences. If you are copying our sample text, replace any instances of XXX with the appropriate details.**<br><br>• If the data are **held or will be held in a public repository**, include URLs, accession numbers or DOIs. If this information will only be available after acceptance, indicate this by ticking the box below. For example: *All XXX files are available from the XXX database (accession number(s) XXX, XXX.*).<br>• If the data are all contained **within the manuscript and/or Supporting Information files**, enter the following: *All relevant data are within the manuscript and its Supporting Information files.*	All relevant data are within the manuscript and its Supporting Information files.

• If neither of these applies but you are able to provide **details of access elsewhere**, with or without limitations, please do so. For example:<br><br>*Data cannot be shared publicly because of [XXX]. Data are available from the XXX Institutional Data Access / Ethics Committee (contact via XXX) for researchers who meet the criteria for access to confidential data.*<br><br>*The data underlying the results presented in the study are available from (include the name of the third party and contact information or URL).*<br>• This text is appropriate if the data are owned by a third party and authors do not have permission to share the data.<br><br>* typeset	
Additional data availability information:	

Manuscript Click here to access/download;Manuscript;Manuscript.docx

Improved Kala-azar Case Management through Implementation of Health Facility-Based Sentinel Sites Surveillance System in Bihar, India

Siddiqui NA, Bhunia GS, Pandey K, Sinha SK, Ansari Z, Topno RK, Lal CS, Ranjan A, Singh VP, Das P, Das VNR*

*Corresponding author

Dr. VNR Das, Scientist-G,
Department of Clinical Medicine,
ICMR-Rajendra Memorial Research Institute of Medical Sciences,
Agamkuan, Patna – 800 007, Bihar, India.
Email ID: drvnrdas@yahoo.com;
Contact no.: +91 9431646332
+91 612 2631565
Fax no.: +91 612 2634379

Abstract

Background

Visceral leishmaniasis (VL), also known as kala-azar, is a neglected vector-borne disease, targeted for elimination, but several affected blocks of Bihar are posing challenge with the high incidence of cases, and moreover the disease is spreading in newer areas. High quality kala-azar surveillance in India, always pose great concern. The complete and accurate patient level data is critical for the current HMIS. On the other side, no accurate data on the burden of Post Kala-azar Dermal Leishmaniasis (PKDL) and co-infections are available under current surveillance system, which might emerge as a serious concern. Additionally, in low case scenario, sentinel surveillance may be useful in addressing post-elimination activities and sustaining KA elimination. Health facility-based sentinel site surveillance system has been proposed, first time, under the present study to do a proper accounting of VL, PKDL and co-infection morbidity, mortality, diagnosis, case management, hotspot identification and monitoring the impact of elimination interventions.

Methodology/Principal Findings

Sentinel site kala-azar surveillance was established and activated at thirteen health facilities in Bihar, using stratified sampling technique during 2011 to 2014. Data were collected through specially designed proforma from all patients attending the outpatient departments of sentinel sites. Among 20968 symptomatic cases attended sentinel sites, 2996 cases of KA and 53 cases of PKDL were registered from 889 endemic villages. The proportion of new and old cases was 86.1% and 13.9% respectively. A statistically significant difference was observed for reduction in KA incidence from 4.13/10000 in 2011 to 1.75/10000 in 2014 ($p<0.001$). There were significant increase (0.08, 0.10 per 10 000 population) in the incidences of PKDL and co-

infection respectively in the year 2014 as compared to that of 2011 (0.03, 0.06 per 10 000 population). The proportion of HIV-VL co-infection was significantly higher (1.6%; $p<0.05$) as compared to other co-infections. Proportions of male in all age groups were higher and found statistically significant (Chi-square test = 7.6; *P=0.026*).Utilization of laboratory services was greatly improved. Friedman test showed statistically significant difference between sensitivity of different anti kala-azar drugs (F = 25.0, P=0.004).The initial and final cure rate of AmBisome was found excellent (100%). The results of the signed rank sum test showed significant symmetry of unresponsiveness rate (*P=0.03*). Similarly, relapse rate of SAG was also found significantly higher as compared to other drugs (95%CI 0.2165 to 19.7035; *P=0.03*). A statistically significant difference was found ($p<0.001$) between villages having 1-2 cases (74%) and villages with 3-5 cases (15%). Significantly higher proportion (95%) of cases were captured by Govt. surveillance (HMIS) ($p<0.001$), as compared to private providers (5%).

Conclusions/Significance

Establishment of a sentinel site based kala-azar surveillance system in Bihar, India effectively detected the rising trend of PKDL and co-infections and captured complete and accurate patient level data. Further, this system may provide a model for improving laboratory services and KA, PKDL and co-infection case management in other health facilities in Bihar without further referral. Program Managers may use these results for evaluating program's effectiveness and improving program management. It may provide an example for changing the practices of health care workers in Bihar and set a bench mark of high quality surveillance data in a resource limited setting. However, generalizability of this sentinel surveillance finding to other context remains a major limitation of this study.

Key words: Kala-azar, Visceral leishmaniasis, Sentinel site surveillance, Post kala-azar dermal leishmaniasis, Co-infection, Hotspot, Kala-azar elimination, Case management.

Author Summary

Visceral leishmaniasis is a neglected vector-borne disease, and one of the major public health problems of Bihar. Disease has been targeted for elimination by 2020. Bihar still posing challenge i.e. incidence is much high in good number of affected blocks, and even the disease is spreading in newer areas. None availability of an accurate data on the burden of Post Kala-azar Dermal Leishmaniasis (PKDL) and co-infections under the current surveillance system is emerging as a serious concern. Therefore, heath facility-based sentinel site surveillance system has been attempted for the first time in Bihar for proper accounting of VL, PKDL and co-infection morbidity, mortality, diagnosis, case management, hotspot identification and monitoring the impact of elimination interventions. A system for capturing regional transmission is essential that can target focal areas of infection to monitor progress towards kala-azar elimination. Sentinel site kala-azar surveillance was established and activated at thirteen health facilities in Bihar during 2011 to 2014. The results of present study showed significant increase in PKDL and co-infection in 2014 when compared to 2011. This further showed that utilization of laboratory services and case management were greatly improved, as majority of patients with VL, PKDL & Co-infections were managed by the sentinel sites itself. The final cure rate of AmBisome was found excellent (100%). These observations may be useful to provide the basis for the design, refinement and resource allocation of the kala-azar control program. This system may also be useful in measuring impact of elimination interventions, their effectiveness and finally help in improving program management. It may further be used as an example for changing the practices of health care workers in Bihar and lesson to how achieve high quality

surveillance data in a resource limited setting. Standardization of sentinel site surveillance in terms of improved procedure, training, logistics, etc may strengthen the effectiveness of this system. Finally, it may possibly be used as a cost-effective approach for capturing real-time kala-azar data in national kala-azar elimination programme.

Introduction

Visceral leishmaniasis (VL) is a neglected vector-borne disease caused by *Leishmania donovani* parasite and is transmitted by the bite of infected *Phlebotomus argentipes* sandflies. The annual burden of VL cases worldwide is 0.2±0.4 million per year. The majority of the cases occur in five countries viz. India, Bangladesh, Sudan, Ethiopia and Brazil. Out of the worldwide VL cases, about 50% reported from India alone, however, Bihar state contributes 80% of VL cases. Most of the endemic population is poor from rural areas living in mud houses[1]. It is suspected to be underestimated by the routine health programme statistics in the absence of a robust surveillance mechanism[2,3,4]. In Bihar, India leishmaniasis manifest in two forms i.e. VL & PKDL. Post Kala-azar Dermal Leishmaniasis is a secondary complication of visceral leishmaniasis (VL) and non-fatal[5]. The disease has been targeted for elimination (reducing incidence to less than one per 10,000 populations at sub-district level by 2020) in the Indian sub-continent due to availability of field based diagnostics (rK-39 strip test) and effective drugs[6]. Surveillance is a key component for any disease control programme but unfortunately in India, kala-azar surveillance is weak. Few patients still seek treatment in the private sector and are not tracked for treatment, efficacy and compliance in the Health Management Information Services (HMIS). Effective Kala-azar surveillance may help in program planning and management. It further informs governments and donors about progress towards Kala-azar elimination. These may be helpful for the design, refinement and resource allocation of control programs[7&8]. Kala-

azar elimination program in India mainly depend on routinely collected health facility-based data through HMIS. The data collected at health facilities vary widely and are subject to bias. There are uncertainties, that whether a patient with kala-azar has been captured by this system correctly. The disease burden of PKDL and co-infections are not well known under current surveillance system. These are emerging as a serious concern and are reported from 36 endemic countries including Bihar, India. There is a need to establish strong surveillance for both the conditions including kala-azar. On the other end, active case detection would become important to kala-azar elimination. Currently, kala-azar fortnights are being organized for active case detection at village level, but it is very expensive and resource intensive.

The current capacity of routine VL surveillance is inadequate and indicators are not properly defined. The quality of data reporting and completeness are also not adequately addressed, however, timeliness reporting is also variable. The capacity of data analysis, data interpretation, and action are extremely limited. The diagnosis of PKDL and co-infections is of variable quality or absent as in the case of malaria[9&10]. However, disease surveillance is considered as one of the principal activity of public health systems. It is always expansive and time taking activity[11]. Kala-azar case reporting is present in endemic districts across the state. A provision of accurate and timely VL surveillance is essential for elimination as in the case of malaria[11]. A well defined system is much required to document regional transmission to monitor progress of kala-azar elimination programme. An effective surveillance system is warranted to target focal areas of infection, and increase capacity to identify hotspots of disease transmission. This may be helpful in monitoring real-time kala-azar data and in identifying changes in kala-azar transmission, morbidity and mortality.

Sentinel surveillance approach for any disease may be useful for early detection of outbreaks, measuring disease burden, change in morbidity, mortality patterns and for timely implementation of control and preventive measures. This is a system in which a designated group of reporting sources such as hospitals; agencies agree to report all cases of one or more notifiable conditions. Primarily, heath facility-based sentinel site surveillance system may be useful to obtained disease trends and improving case management. Sentinel surveillance offer greater design flexibility as in case of malaria and tuberculosis, when compared to population-based surveillance[12&13]. Although results obtained through sentinel surveillance may not be representative of the entire population but it may be useful in reflecting the trends based on accurate assessment. Similar studies on kala-azar are not available in Bihar. The aim of present study was to describe the design and implementation of kala-azar surveillance and its utilization for epidemic detection and response. Further, it may be used for programme decision making. Data collected under this study may fill the gap of existing HMIS and reassess the prioritization of resources and to validate the information emerging from the health management information system (HMIS). Further, it may be useful in measuring impact of elimination interventions. To the best of our knowledge, this is the first study of its type from Bihar on kala-azar.

Materials and methods

Study area and selection of sentinel sites

In Bihar, twenty one (21) districts were identified as high endemic for kala-azar for the year 2010, reporting on an average 1800 cases, in a range of 208-4660 cases. The incidence proportion ranged from 1 to 25 per 10 000 persons with an average of 7 per 10 000 persons. All these districts situated mainly to north of the river Ganges. Nearly 80% of the total cases reported by the programme were from 12 districts of Bihar. These districts are East Champaran,

Sitamarhi, Madhubani, Darbhanga, Muzaffarpur, Samastipur, Vaishali, Saran, Begusarai, Saharsa, Madhepura and Purnia. Sentinel sites were made in 4 high endemic districts of Bihar, namely, Muzaffarpur, Samastipur, East Champaran and Saran as per their incidence proportion rate (IPR). These districts were selected because of piloting of various interventions under existing Kala-azar Elimination Program, and on the basis of the geographical locations. The districts were selected from all the direction. The due consideration has been given to Program commitment for making provision of rapid diagnostic test kits (rK-39) and the first line anti Kala-azar drug i.e. miltefosine in the selected study districts. In each district, two Primary Health Centres (PHC's) were selected as sentinel sites again as per the incidence proportion rate (IPR) (**Figure 1**). Additionally, one district hospital from each district was also selected as sentinel site. One medical college, located at Muzaffarpur was also selected as sentinel site. Thus the present study was conducted in thirteen sentinel sites.

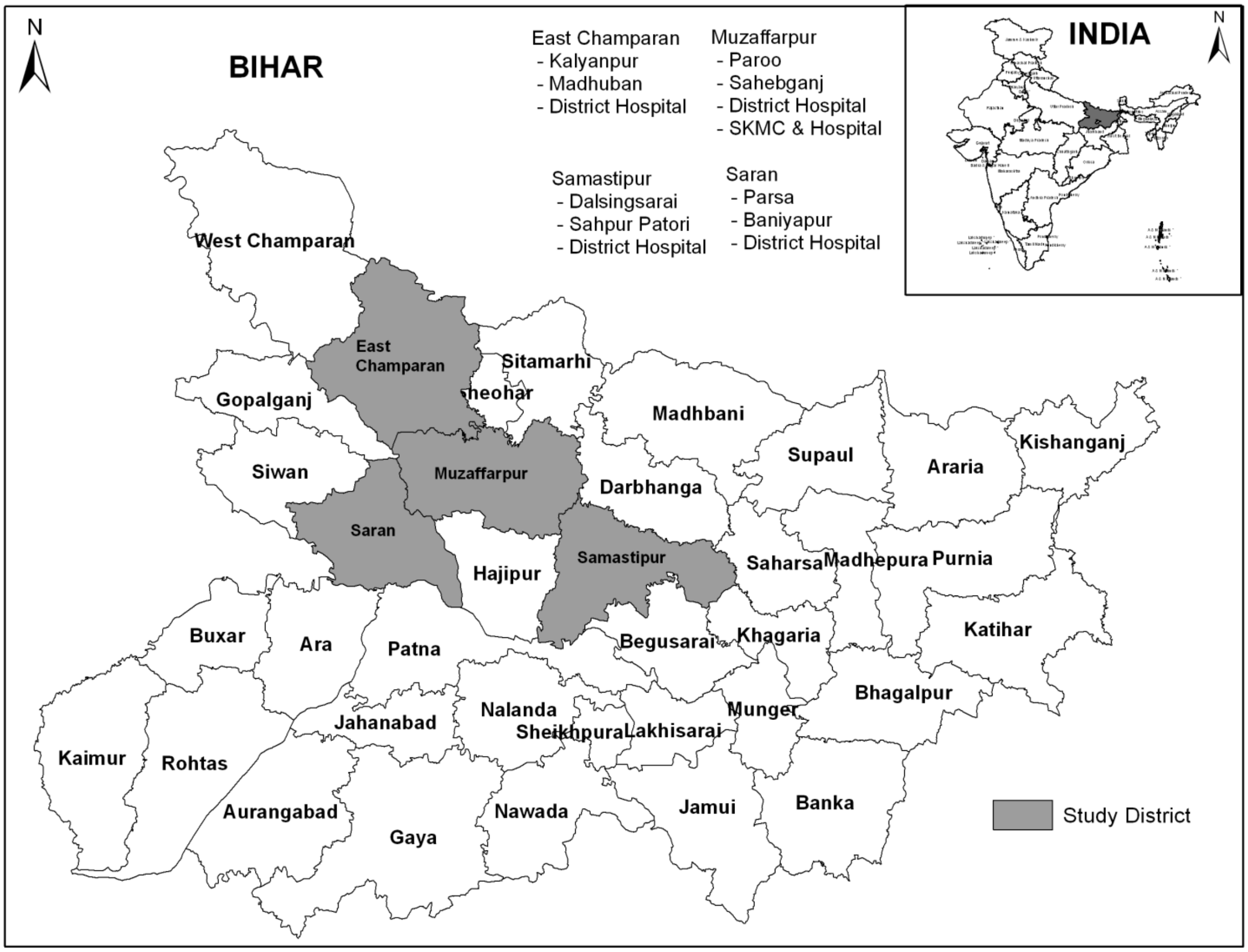

Figure 1 Location map of the study area

Launching of health facility-based sentinel sites

Rajendra Memorial Research Institute of Medical Sciences (RMRIMS) in collaboration with National Vector Borne Diseases Control Programme (NVBDCP), State Program office as well as District Program office, Bihar established a health facility based Kala-azar sentinel surveillance sites. These sentinel sites were selected as per the fulfillment of readiness criteria viz.

i. Availability of adequate and appropriate staff at the selected sentinel sites.

ii. Trained and competent project staff with good understanding of their roles and responsibilities for the research work.

iii. Motivated health care providers with package of services for Kala-azar.
iv. Consistent availability of diagnostics, drugs, equipment etc.
v. Provision of individual drug packet for each diagnosed Kala-azar case to ensure complete treatment.
vi. Availability of treatment cards and d its accurate filling by the health care providers at the study site.
vii. Regular motivational activities for Kala-azar patients by the health workers to complete their treatment.
viii. Operational follow up mechanisms in place at the site.

Deficiencies of all selected sentinel sites were identified, and efforts were made to improve all the sentinel sites to meet the readiness criteria within stipulated times (3-6 months). Research activities were started after meeting readiness criteria at the site.

Sample size

Keeping the criteria of selection of sentinel site, fulfillment of readiness criteria for health facility based sentinel sites and resources in mind; the sample size for this study was restricted to 13 sentinel sites.

Sampling technique

Study districts were selected on the basis of piloting of various interventions under existing kala-azar Elimination Program, and on the basis of the geographical locations. In addition, the due consideration has been given to Program commitment for making provision of rapid diagnostic test kits (rK-39) and the first line anti kala-azar drug i.e. miltefosine in the selected study

districts. The stratified sampling technique was attempted for the selection of sentinel sites according to the incidence proportion rate (IPR) of PHC's.

Study duration

The present study was conducted during the period 2011 to 2014.

Recruitment of personnel for sentinel sites

Four Site Supervisors (Research Officer) with post-graduate qualification and experience of working in public health were appointed for all the study districts. The work of one district was assigned to one site supervisor. These site supervisors were imparted extensive two-weeks training at RMRI related to Kala-azar management and surveillance techniques.

Training of site supervisors and quality assurance

Staff including site supervisors at the sentinel sites were imparted two weeks extensive training covering all important aspects of kala-azar in order to enable them to discharge their responsibilities smoothly. Training was organized at RMRI where senior faculty and experts were engaged as the facilitators/trainers. The curriculum and detailed training module in form of written text material and standard operating procedure (SOP) was prepared. Training module was developed by the senior faculties of RMRI in consultation with NVBDCP, keeping in view the desired job responsibilities and activities of the site supervisors and other staff engaged. During the training, all the procedures were taught through hands on training using the formats proposed to be used. Besides, the induction training, RMRI also organized on-site training periodically and regularly throughout the project tenure to the site supervisors and other staff members involved in sentinel sites activities for improving skill and motivating the staff members. They were imparted short-term hands on (recurrent) training on various aspects like

patients card filling, data management, computer skills, data presentation and reporting etc, through hands on practice under supervision of the senior faculties of RMRI including principal investigator.

Sentinel surveillance network

There were two types of network agencies and functionaries working at different level separately and in close co-ordination with other agencies. First networking worked on the basis of hierarchical system using bottom-to-top approach in terms of responsibilities and accountabilities. A "Memorandum of Understanding (MoU)" was signed by all the participating sentinel sites identified in the surveillance network. The sentinel surveillance limited to level-II and level-III facilities only because of the availability of diagnosis and treatment at these level only. The involvement of level-I facility staff was limited to early detection of VL cases and for follow up of patients, and for regular monitoring of adverse effects. Every sentinel site was assigned the task of completion of treatment for each patient. None of the private health care facility dealing with kala-azar was inducted under the sentinel sites for this study due to lack of evolved cost effective mechanism. The criteria to utilize the services of existing facilities were developed by the site supervisors with the help of kala-azar Technical Supervisors (KTS) and Vector Borne Diseases Consultants (VBD's) of the district. RMRI was taken special effort to ensure that these centers must satisfy all the readiness criteria and comply with the norms and standard required for a sentinel site, before signing MoU. After the signing of memorandum of understanding the staff was imparted short training for filling up the patient cards and keeping the records complete in terms of treatment, side effects and follow-up to assess the effectiveness of drugs used for treatment.

Case definition, diagnosis and case management

A standard protocol for the case definition of KA, PKDL, Co-infection, and severe case of KA, diagnosis, and case management was adopted by all the sites under the surveillance network for maintaining uniformity in recording and reporting across the sentinel sites.

Case Definition of Kala-azar

An individual with fever of more than 15 days duration with splenomegaly and not responding to anti-malarial drugs and found positive by rapid diagnostic blood test (rK-39 strip test), or a case confirmed with laboratory-based diagnosis i.e. splenic/ bone marrow aspirate examination for *Leishmania donovani* (L.D).

Case Definition of Post Kala-azar Dermal Leishmaniasis (PKDL)

An individual with or without past history of Kala-azar presented with maculo-papular or nodular lesions with rK-39 test positive, confirmed by slit-skin smear / skin-biopsy examination for presence of parasites was labeled as PKDL case.

Case Definition of Co-infection

A confirm case of kala-azar with HIV/TB positive test result was declared as co-infection.

Severe Case of Kala-azar

The definitions used for severe cases of kala-azar were:

- Resistant and relapse cases of VL.
- Kala-azar with severe anemia (Hemoglobin less than 5 g/dL).
- Kala-azar patient suffering from another serious disease like renal and hepatic conditions.

Diagnosis of KA and PKDL

A standard protocol for the diagnosis of VL and PKDL was followed by all sentinel sites. The case of kala-azar was either diagnosed by positive test result of rapid diagnostic strip test (rK-39) or with laboratory-based diagnosis i.e. splenic/ bone marrow aspirate examination for *Leishmania donovani* (L.D). PKDL case was diagnosed by rK-39 test positive, and by slit-skin smear / skin-biopsy examination for presence of parasites. The rapid diagnostic test kit (rK-39) of kala azar and PKDL was made available to all sentinel sites by the site supervisor. Hospitals and doctors located at level-2 and level-3 was approached for the participation in the surveillance by the site supervisors. In case of shortage of rapid diagnostic test kit (rK-39) at the site during the study, it was provided under the sentinel program. Training was imparted to doctors, health workers and laboratory technicians of sentinel sites for managing rK-39 strip test. Quality assurance of the dipstick test was carried out periodically to maintain the consistency of the test.

Diagnosis of co-infection and severe cases

Co-infection cases were diagnosed with the help of sputum for AFB/RDT for tuberculosis, X-ray chest, ELISA (TB) and ELISA (HIV). Additional tests viz. hematogram, biochemical, etc. examinations were also done to capture co-infection. However, such cases were finally referred to RMRIMS for diagnosis and treatment.

Case management of KA and PKDL

All the sentinel sites followed a standard protocol of the treatment for Kala-azar and PKDL. Treatment of kala-azar and PKDL was provided after admitting the patient at sentinel sites. The complete information of hospitalized cases was recorded in the patient treatment card. Treatment cards were in placed at every sentinel sites. Vital information on the place, age, sex, marital

status and pregnancy and its outcome was filled up in the treatment card. Additionally, information about drug used for treatment of VL & PKDL, treatment duration, treatment status, adverse events of drugs, reasons of treatment discontinuation of treatment other than side effects of drug, etc., were also collected.

Site Supervisors in each district motivated the treating physicians and other staff members to fill up the form completely. Miltefosine was preferred first line drug of kala-azar during the study period. It was used for treatment, except in woman during first trimester of pregnancy, married women of child bearing age who are not using contraceptives regularly and are at risk of becoming pregnant. Miltefosine was not used for treatment in case of breast feeding mother and infants. Arrangements were made for doing a pregnancy test to ensure that married women in reproductive age and not using contraceptives to rule the pregnancy at all sentinel sites. A woman found positive for pregnancy, was treated with other drugs as recommended in the programme. The women in child bearing age were provided adequate contraception during the treatment and till six months after the treatment and during follow up. The local antenatal clinics located at the sites were asked to supply contraception to child-bearing age women. In addition, a pregnancy register was created at each sentinel sites for keeping the record of diagnosis, treatment and follow-up with regard to drug compliance, adverse effects etc. of VL pregnant women. A separate database for keeping record of women in child bearing age and lactating mother reporting to sentinel sites were prepared and utilized. Cases of severe under-nutrition, severe anemia and patients with known history of kidney or liver disease were not being treated with miltefosine. The data was collected at each site with the help of SOP, used for filling various formats to ensure the quality of data. Filled up patient treatment cards of Kala-azar and PKDL were collected by site supervisor at each site. The filled up form were checked and

verified by the site supervisors who filled the pre-designed template form and create patient database for their respective districts. Line listing of Kala-azar, PKDL and disaggregated reports were generated every month using database.

In case of death in course of treatment and follow-up, causes of death were recorded by the site supervisor with the assistance of the treating physicians. The causes of death were investigated thoroughly in order to assess the specific cause of deaths such as drug intolerance, toxicity, cardiac-arrest and other reasons of deaths in details. The classification of death after patient review and audit was identified as a death attributable to kala-azar or death that caused with an associated illness. A list of patients who died during the course of treatment or follow up was prepared monthly. The treating physicians at each sentinel sites was directed to certify and fill-in the cause of death column in the patient treatment card properly. In case of those patients who left the hospital occasionally for different reasons follow up mechanism was developed and in case of death an investigator visit the home of the patient, efforts were taken to determine the cause of death based on the information collected from the health workers such as ANMs, ASHA, paramedical staff, etc. The follow up of those patients were also done whose death information were not recorded in the hospital. In case of cause of death were not mentioned properly, verbal autopsy of a close relative of patient were conducted by the site supervisors and study coordinator by visiting the houses of persons with death due to kala-azar. Verbal autopsy was performed by a trained person. Village health workers and volunteers engaged for this project working at sub-PHCs were instructed to report every death that occurred in their areas of monitoring to the sentinel site supervisors. RMRIMS constituted a panel of experts to review the report of the verbal autopsy conducted by the site supervisors and coordinator. In any circumstances, cause of death was finally decided by the panel of expert constituted for the

purpose. Sentinel sites adopted above defined protocol for setting an example to others, not functioning under surveillance network. The information on verbal autopsy of the cases not admitted to any hospital were tracked and reported separately. Kala-azar patients admitted to the sentinel sites were used for estimation of case fatality rates of the disease.

Case management of co-infection

Treatment of co-infections was provided as per the guidelines of NVBDCP, AIDS control programme and TB control programme. The sentinel sites had no networking with institutions / facilities in the district to capture information on HIV-VL co-infections. The testing for HIV of kala-azar patients at high risk was performed at district level. However, such cases were finally referred to RMRIMS for final diagnosis and treatment.

Case management of severe cases of KA

Severe anemic patients were treated with blood transfusion and when haemoglobin raised by medicines (iron and folic acid), specific treatment was started. In case of delayed treatment, patients were treated with either amphotericin-B or AmBisome®. Similarly, for undernourished cases efforts were taken to improve the nutritional status of the patient with kala-azar before administration of specific treatment. The cause of death in case of severe case of kala-azar was also investigated thoroughly by the site supervisors in consultation with the treating physicians at the sentinel sites. The causes of death were specified categorically so that mortality due to VL was assessed. On the basis of that, overall mortality rate of VL per sentinel sites were ascertained.

Data Management and Information System

Records of all the patients visiting and enrolled for treatment at the sentinel sites were collected in a pre-designed format. All the formats used under the study were pre-tested and modified accordingly. Site supervisors collected completely filled in patient cards and other records for all the patients. Checking and verification of all filled in patient cards/records to maintain the consistency in data were performed by coordinator of the study. Incomplete patient's card and errors found in cards were re-checked and verified with the assistance of the physicians at the sentinel sites for immediate corrections. Completely filled in and checked cards were transferred to the computer using a pre-designed electronic template for creating database for each site and handed a copy of the monthly data to the district program managers such as VBD Consultant and District VBD Officer. Checking and verification of the entire data sheet in the database was done by District VBD Consultant at district level and feedback on report if any was sent to site supervisor present in the district. The site supervisors were also trained to analyze and disseminate the data in form of graphics and reports to higher levels. At the same time, information for action at each level was used for feedback and for capacity development, which helps in improving the services rendered to the patient. All the information from each of the sentinel sites were shared with RMRI. RMRI consolidates the data and generate the report which sent to all higher levels in the existing system and feedbacks from higher levels were received, taken into consideration for improvement in future report. Every effort was taken to strengthen the information collected and thus to improve existing surveillance system. Special efforts were taken to monitor the quality of data generated at these sentinel sites. A copy of the monthly report was sent by site supervisor to District VBD Officers for appropriate action and to provide feedback on monthly report. A consolidated report from sentinel sites pertaining to the major

achievements for kala-azar were prepared at the end of every quarter and shared to higher levels for their feedbacks to strengthen the existing surveillance system under the program.

Surveillance procedure

Sentinel surveillance used various approaches for collecting information about all types of kala-azar patients. In first year of the study, usual passive case reporting method was adopted in all sentinel sites and patient treatment card was used for collecting information. Site Supervisors prepared line listing of kala-azar cases by village/by areas for each month at the end of first year. Thus clusters of high incidence villages/areas of kala-azar were identified on the basis of the line listing and finally hot spots for kala-azar were recorded and documented. Similarly, kala-azar cases reported to the district hospitals/medical college at district level through passive case detection were compiled (line listed) for every month. Finally at the end of the year of surveillance, line listing available at the site was matched/cross verified from the office of District Malaria Officers of respective district. Health workers including ASHA's and volunteers were intimated to monitor adverse events, and treatment compliance for the cases not reported to sentinel sites for follow up.

Reporting Method

The site supervisor prepared a monthly consolidated report in the pre designed format for each kala-azar case. A one-page case report extracted from patient treatment card/other records were generated to see the consistency and completeness of reporting. The information extracted from the records at each level is listed below:

- Number of cases of kala-azar based on 'rK-39'positivity
- Number of cases diagnosed by microscopy

- Drug used for the treatment
- Number of cases completed the treatment
- Number of cases dropped out before completing the treatment
- Number of cases not responded to treatment
- Number of cases admitted to the hospital
- Number of cases died in the hospital
- Number of cases died at home
- Number of patients developed major side effects of drugs

Line listing of every Kala-azar and PKDL cases were maintained at all sentinel sites. Reporting was done through line listing process to supplement the existing reporting system.

Ethical statement

The study was duly approved by the Rajendra Memorial Research Institute of Medical Sciences, Ethics Committee and the Scientific Advisory Committee. A written informed consent was obtained from the head of all sentinel sites. A written informed consent was also obtained from the subjects admitted for treatment at sites. Subjects <18 years old who were able to read provided written assent following written consent from their parent or guardian. All study data were anonymized before analysis. Confidentiality was maintained in full compliance with the principles of the Declaration of Helsinki (as amended in Tokyo, Hong Kong, and Somerset West, South Africa).

Statistical analysis

Descriptive statistics of the data were calculated. Proportions of patients and demographic characteristics were appropriately prescribed. Incidence rates of the disease were calculated per10, 000 inhabitants. The chi-square and Wilcoxon tests were performed to test the statistical significance of differences in a classification system. The Bland-Altman plot (Bland and Altman, 1999) was adopted to compare two measurements techniques. One-sampled t-test was used based on logarithmic transformation to measure the significant differences of efficacies of drugs used for kala-azar treatment. Freidman test (Conover, 1999) was used to assess the difference between sensitivity of different anti-kala-azar drugs. The Signed rank sum test was performed to measure the symmetrical differences between the sample median and the test value. All the analysis were performed at <0.05 significance level.

Results

A total of 20968 symptomatic cases (fever more than 15 days) were screened and out of that, 2996 confirmed cases of KA and 53 cases of PKDL, total (3049) were registered from 2011 to 2014 in all the sentinel sites. The percentage share of male and female was 56.4% and 43.6% respectively. Sentinel Sites covered a total of 889 endemic villages. A statistically significant difference was found ($p<0.001$) between villages having 1-2 cases (74%) and villages with 3-5 cases (15%). Good proportion (4%) of villages was still with more than 10 cases. The overall proportion of kala-azar cases were 14.29% in the screened cases. The Baniyapur (Saran) site contributed highest proportion (35.78%), while lowest (8.67%) by Kalyanpur site (East Champaran). The proportion of new cases was 86.1% amongst all cases registered under the sentinel sites while 13.9% cases were old cases. Amongst old cases, 4% cases were unresponsive and 9% were relapsed. About half of the VL (47%) cases were in the age-group 16-44 years.

Overall, decreasing trends of incidence per 10 000 population was noticed from 2011 to 2014 for all sites. Kala-azar was significantly reduced by more than 50% from 4.13/10 000 to 1.75/10 000 with mean 2.61 and 95% CI (2.26-3.41) for the study period. Highest mean incidence (4.71/10000) was recorded for Madhuban PHC (East Champaran) with 95% CI as 3.77-6.88. In 2014, about (38%) sites reached to elimination target (<1case per 10000 populations). However, Baniyapur site (Saran) showed alarming situation (4.11 per 10000) in 2014 as compared to 2011 (5.63). Overall, mean value of the incidence was recorded 2.61 with 95% CI as 2.26 to 3.41.

Table-1

Table-1: Site wise kala-azar incidence/10,000 population

**Sentinel sites**	**2011**	**2012**	**2013**	**2014**	**Mean**	**Median**	***CI***
**East Champaran**							
District Hospital	3.53	2.45	1.27	0.96	2.05	1.86	1.70 - 2.87
Kalyanpur PHC	3.10	3.31	1.73	1.41	2.39	2.42	2.10 - 3.06
Madhuban PHC	9.01	4.82	3.16	1.86	4.71	3.99	3.77 - 6.88
**Muzaffarpur**							
Medical College (SKMCH)	1.15	2.14	0.87	2.40	1.64	1.65	1.42 - 2.16
District Hospital	2.31	6.86	2.90	1.60	3.42	2.61	2.71 - 5.06

Paroo PHC	6.77	2.76	2.52	2.38	3.61	2.64	2.97 - 5.08
Sahibganj PHC	5.55	3.26	2.26	1.01	3.02	2.76	2.44 - 4.36
**Samastipur**							
District Hospital	0.35	0.21	0.10	0.85	0.38	0.28	0.28 - 0.61
Dalsinghsarai PHC	8.49	3.85	1.34	1.76	3.86	2.81	2.87 - 6.15
Shahpur Patori PHC	2.78	1.46	0.74	0.93	1.48	1.20	1.20 - 2.12
**Saran**							
Sadar Hospital, Saran	1.17	1.87	1.16	1.05	1.31	1.17	1.20 - 1.57
Baniyapur PHC	5.63	2.68	2.74	4.11	3.79	3.43	3.37 - 4.76
Parsa PHC	6.29	4.27	1.23	2.47	3.57	3.37	2.90 - 5.10
**Total**	**4.13**	**2.88**	**1.67**	**1.75**	**2.61**	**2.32**	**2.26 - 3.41**

Monthly distributions of disease showed higher (11.2%, 11.7%, 10.0% and 8.5%) incidences for the months of January, March, July and August respectively for every year. However, it was lesser (5.8%, 6.1%, 6.7%) for the month of April, September and November.

There was significant increase (0.08, 0.10 per 10 000 population) in PKDL and co-infection respectively in 2014 when compared to 2011 (0.03, 0.06 per 10 000 population) **Figure-1**.

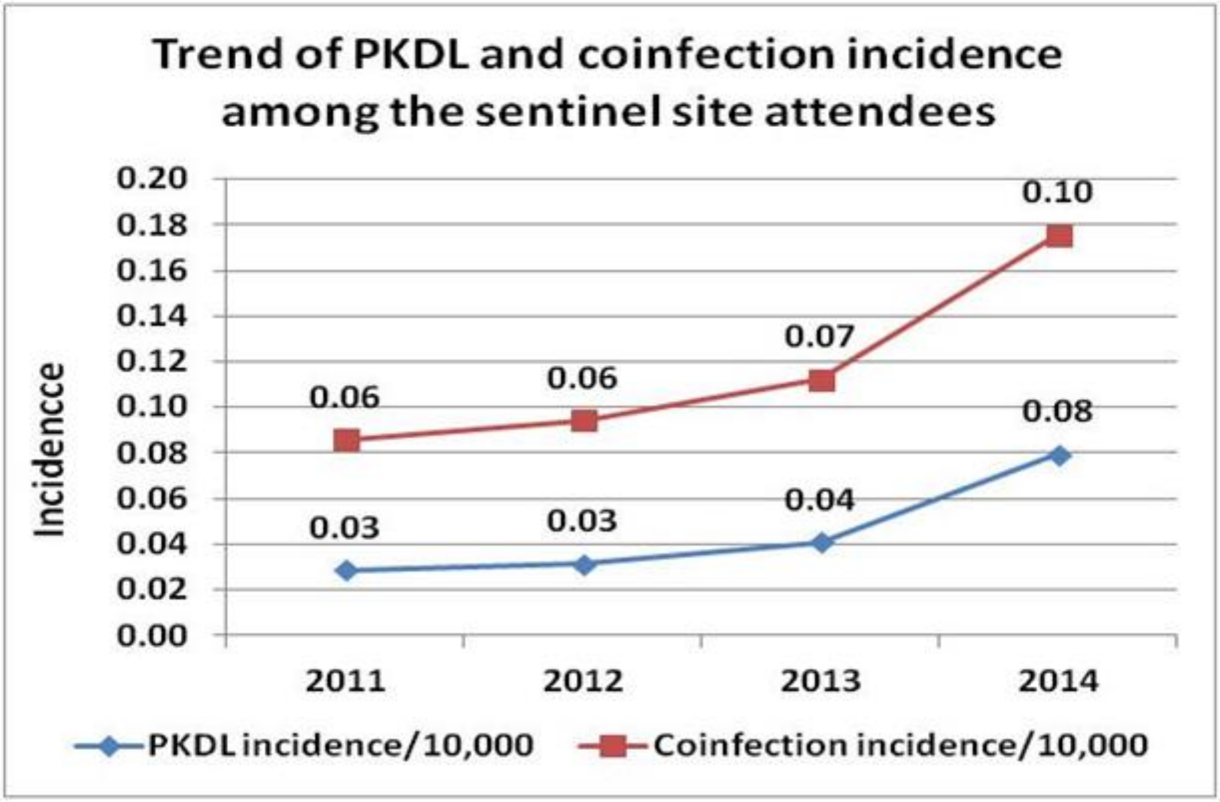

Figure-2: Trends of PKDL and co-infection incidence among the sentinel site attendees

Analysis of district-wise trends of PKDL, showed overall rising trend in almost all the districts except East Champaran for the study period. A sharp increase in number of PKDL cases was observed for Saran district in 2014 when compared to 2013. The number of PKDL cases was almost double in Muzaffarpur and Samastipur district in the year 2013 when compared to 2011. On the whole co-infection was also found almost double (29) in 2014 when compared to 2011 (16), and this difference was statistically significant ($p<0.05$), which showed rising trends in cases of co-infections. In East Champaran district, co-infection was found very alarming i.e. more than 200% times increased in number of co-infection cases from 2011 to 2014. The overall

proportion of co-infection and PKDL were 2.80% and 1.74% respectively. The demographic distribution of overall illness revealed that overall; VL plus co-infection were slightly higher (55.6%) for males as compared to females (44.4%). Similarly, proportion of PKDL was also higher (52.8%) for males as compared to females (47.2%). The prevalence of co-infection was recorded highest (3.34%) for the age-group 16-44 years. Majority of the cases (87%) were from rural areas. In total, 84 cases were identified as with concurrent illness (co-infection) and proportion of kala-azar cases with concurrent illness was 2.80%. Out of that, majority (56%) were suffering from HIV-VL co-infection, followed by (13%) TB-VL co-infection. The liver disease also contributed significantly (8%). The proportion of HIV-VL co-infection was significantly higher (1.6%; $p<0.05$) as compared to other co-infection. As regard to fever history of kala-azar cases, significantly higher proportion (60.6%) was with fever between 15-30 days, followed by (32.5%) with 30-45 days of fever. Analysis further revealed that, the patients having fever of longer duration (> 30 days) was with significant weight loss. Significantly higher proportion (61%) of kala-azar cases was with 2-3 cm spleen size when compared with 3-4 cm (about 18%) at the time of diagnosis. This difference was found statistically significant ($p<0.05$). However, larger spleen (4-6 cm) size was also noticed for only negligible proportion. Out of the female patients (1329), about 47% were from reproductive age-group. Almost, 50% of the reproductive age group patients were from most sensitive child bearing age group (21-30). Amongst the reproductive age-group patients (625), 3.7% (23) were found pregnant. None of the pregnant woman encountered with teratogenic effect in the delivered child.

Demographic characteristics of the sentinel sites

Analysis further revealed that maximum cases (47%) were in the age group of 16 – 44 years followed by (35%) in the age group of ≤ 15 years. Proportions of male in all age groups were

higher and found statistically significant (Chi-square test = 7.6; *P=0.026*). Percentage of kala-azar cases was found in decreasing order right from secondary level to post graduation level education. Results revealed that significantly higher proportion (69%) of kala-azar cases were from up to middle level of education, and out of that, 33% were illiterate ones. Significantly lower proportion (only 9%) of kala-azar cases were from graduation and above level of education status. The educational status between male and female categories were also found statistically significant (Wilcoxon test; P=*0.007*). About, 48% of kala-azar cases were from low socio-economic (agricultural laborer & laborer) groups. A very negligible proportion (9%) of kala-azar cases were from upper socio-economic groups. Good proportions (7%) of cases were from the children below 6 years and aged individuals. The male and female categories among the different occupational groups showed statistically significant (*P<0.001*) result. Overall, significantly higher proportion (51%) of kala-azar cases were from schedule caste categories, followed by (33%) from Other Backward Caste (OBC) categories as compared to General caste categories (16%). Social category between male and female groups were not found statistically significant (*P=0.77*). **Table-2**

Table-2: Demographic characteristics of kala-azar cases by sites (N=2996, Male=1667; Female=1329)

**Variable**	**Category**	**Male**		**Female**		**Total**	
		**(N)**	**(%)**	**(N)**	**(%)**	**(N)**	**(%)**
**Age**	≤ 15	560	33.6	489	36.8	1049	35.0
	16 – 44	781	46.9	627	47.2	1408	47.0

	≥ 45	326	19.6	213	16.0	539	18.0
**Educational status**	Illiterate	553	33.2	434	32.7	987	32.9
	Literate	119	7.1	108	8.1	227	7.6
	Primary	292	17.5	264	19.9	556	18.6
	Middle	176	10.6	116	8.7	292	9.8
	Secondary	214	12.8	176	13.2	390	13.0
	Higher Secondary	137	8.2	129	9.7	266	8.9
	Graduation	109	6.5	79	5.9	188	6.3
	Post-Graduation & above	67	4.0	23	1.7	90	3.0
**Occupational Status**	Farmer	176	10.6	92	6.9	268	9.0
	Agricultural Labor	455	27.3	363	27.3	818	27.3
	Laborer	458	27.5	156	11.7	614	20.5
	Business	94	5.6	23	1.7	117	3.9
	Service	62	3.7	95	7.2	157	5.2
	Students	304	18.2	232	17.5	536	17.9
	Housewife	0	0.0	281	21.1	281	9.4

	None	118	7.1	87	6.6	205	6.8
**Social Category**	General	277	57.9	201	42.1	478	15.9
	OBC	566	56.4	437	43.6	1003	33.5
	SC	824	54.4	691	45.6	1515	50.6

The Bland-Altman plot was used to measure the relationship between the differences and the averages of male and female group to look for any systematic biases and to demarcate any possible outliers between demographic characteristics in the sentinel sites. **Figure-3**

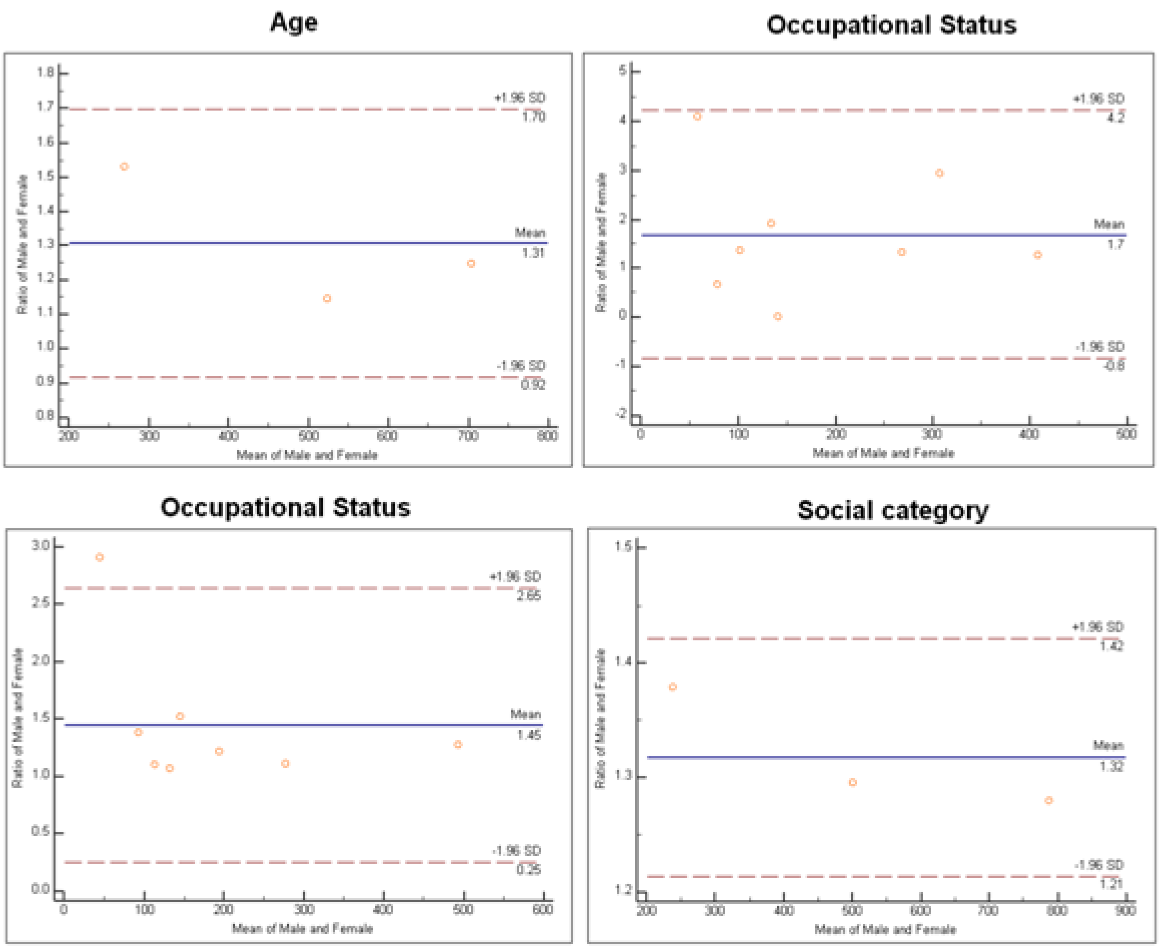

Figure-3 Bland-Altman plot of demographic characteristics of the Sentinel sites

Diagnostic analysis

A total of 20,968 potentially suspected individuals, comprising 11285 males and 9683 females were screened for kala-azar with rK-39 rapid strip test. Out of that, 3049 (56.4% males and 43.6% females) were found rK-39 positive. The overall, percentage of rK-39 positivity was about 14%, consistent in males and females. The rK–39 positivity was slightly higher in the age-group 16-44 years for both the sexes. A total of 608 cases were subjected to different tests to rule out relapse, re-infection, co-infection and PKDL cases. Out of that, 67% were found positive for the different tests. The sensitivity of splenic aspirate was found significantly higher (94.3%), followed by bone marrow (80.1%). Altogether, 53 (1.7%) cases were confirmed as PKDL with

the help of slit-skin smear /skin-biopsy. However, the sensitivity of slit-skin smear / skin-biopsy was found 55.2%. As regard to co-infection, maximum sensitivity was recorded as 42.7% for ELISA (HIV). Utilization of laboratory services was greatly improved.

Table-3

Table-3: Distribution of other diagnostic tests for co-infection, PKDL, relapse and re-infection

**Categories**	**Male**			**Female**			**Total**		
	Total tested	+ve	%	Total tested	+ve	%	**Total tested**	**+ve**	**%**
ELISA (HIV)	63	29	46.0	47	18	38.3	110	47	**42.7**
BM	93	74	79.6	58	47	81.0	151	121	**80.1**
SPA	96	89	92.7	61	59	96.7	157	148	**94.3**
Skin Smear	50	28	56.0	46	25	54.3	96	53	**55.2**
Microscopy/RDT for Malaria	8	2	25.0	5	1	20.0	13	3	**23.1**
Sputum for AFB/RDT for TB	21	8	38.1	14	3	21.4	35	11	**31.4**

Other (Hematogram & Biochemistry)	22	9	40.9	24	14	58.3	46	23	**50.0**
**Total**	**353**	**239**	**67.7**	**255**	**167**	**65.5**	**608**	**406**	**66.8**

Efficacy of anti kala-azar drugs administered at sentinel sites

The higher proportion of kala-azar cases were treated with miltefosine (43.9%) as compared to other drugs viz. amphotericin-B (11.8%), Miltefosine+Paramomycin (5.1%), SAG + Miltefosine (4.8%) and AmBisome (2.5%). Good proportion of kala-azar cases (27.0%) were also treated with SAG. Statistically significant differences in efficacies were observed for SAG (t-test = 6.98; *P<0.0009*), SAG + Miltefosine (t-test = 7.49; *P<0.0007*), Miltefosine (t-test = 5.57; *P<0.002*), Amphotericin B (t-test = 4.46; *P<0.002*), Miltefosine + Paramomycin (t-test = 7.93; *P<0.03*) and Ambisome (t-test = 2.06; *P<0.09*).

Friedman test showed statistically significant difference between sensitivity of different anti Kala-azar drugs (F = 25.0, P<0.004). The initial and final cure rate of Ambisome was found excellent (100%). Whereas, other drugs viz Miltefosine + Paramomycin (combination therapy) had also showed comparable efficacy (100%; initial & 99.3%; final cure) with that of Ambisome. Similarly, the initial and final cure rate of Amphotericin B was also found significantly higher (99.7% & 98% respectively). As far as miltefosine is concerned, final cure rate was also promising (94.1%). The sensitivity of SAG was found very poor in the treatment of kala-azar and a big difference was observed in the initial (88.1%) and final cure rate of SAG (59.3%). **Table-4**

Table 4: Efficacy of drugs administered for the treatment (N=2849)

**Description of drugs**	**Cases treated**	**Initial Cure Rate n(%)**	**Final Cure Rate n(%)**	**Unrespon -siveness Rate n(%)**	**Relapse Rate n(%)**	**LAMA/ Defaulter Rate n(%)**	**Death Rate n(%)**
SAG	808	655 (81.1)	479 (59.3)	153 (18.9)	176 (21.8)	8 (1.0)	10 (1.2)
SAG + Miltefosine	145	142 (97.9)	126 (86.9)	3 (2.1)	16 (11.0)	15 (10.3)	2 (1.4)
Miltefosine	1314	1306 99.4)	1236 (94.1)	8 (0.6)	70 (5.3)	43 (3.3)	12 (0.9)
Amphotericin B	354	353 (99.7)	347 (98.0)	1 (0.3)	6 (1.7)	7 (2.0)	2 (0.6)
Miltefosine + Paramomycin	153	153 (100.0)	152 (99.3)	0 (0.0)	1 (0.7)	0 (0.0)	1 (0.7)
Ambisome	75	75 (100.0)	75 (100.0)	0 (0.0)	0 (0.0)	0 (0.0)	0 (0.0)

#LAMA=73: Treated at RMRI &MSF=127)

The rate of un-responsiveness of SAG was found significantly higher (18.9%) as compared to rest of the drugs, while un-responsiveness rate for SAG + Miltefosine (combination) was found as negligible (2.1%). The results of the signed rank sum test showed significant symmetry of unresponsiveness rate ($P<0.03$). Similarly, relapse rate of SAG was also found significantly higher as compared to other drugs (95%CI 0.2165 to 19.7035; $P<0.03$). There was significant differences of the LAMA/defaulter rate between the anti-kala-azar drugs (95%CI -1.2634 to 6.8634; $P<0.03$). However, no significant differences of death rate were observed between the different anti-kala-azar drugs ($P<0.06$).

ADR

Besides, baseline clinical examination (0 day), the study subjects were clinically examined on each visit to assess clinical improvement as well as AE, if any. Altogether, 401 AEs were reported in the study subjects, of which gastrointestinal side effects, such as nausea (26.4%), diarrhoea (22.2%), abdominal pain (12.7%), and dehydration (9.7%), were the most common recorded complaints. All patients were graded under CTC; 256 (64%) were in grade I, 75 (19%), 52 (13%), seven (1.5%), and 11 (2.5%) were in grades II, III, IV, and V, respectively.

Severe ADR

Of 11 deaths reported as Serious Adverse Event (SAE), the explored cause of deaths were extreme diarrhea (N=7), kala-azar-pulmonary tuberculosis co-infection (N=2), HIV/AIDS co-infection (N=1), and acute pancreatitis (N=1). A total of 33 patients required hospitalization during domiciliary mode of treatment with miltefosine to manage the AEs. In those patients having ADR, 6% were suffering from severe ADRs, 58% were suffering from moderate ADR, whereas the rest were suffering from mild ADRs.

Focused villages of sentinel sites having > 10 cases/year during the study period

Out of 889 endemic villages, only 6 (0.7%) villages under two sentinel sites viz. Kalyanpur and Madhuban of East Champaran district contributed 76 kala-azar cases in every year and thus a total of 304 cases were from these villages. Out of the total cases registered (2996) at sentinel sites, 10% share were from these six highly endemic villages. The highest cases (19) were recorded in Shambu Chak village under Kalyanpur sentinel site of East Champaran district in each year.

Concordance between HMIS and Sentinel sites data

Analysis primarily consists of determining the discordance between the two sources. The cases registered under sentinel sites were compared to that of VL cases captured under existing surveillance system and it may provide preliminary leads to efforts required to strengthen HMIS. Analysis showed that significantly higher proportion (95%) of cases was captured under HMIS. HMIS data almost close to actual disease burden. The similar pattern was noted for every district except Saran district (89.7%). **Table-5**

Table-5: Concordance between Sentinel Surveillance and HMIS data by district

Sl.	District	2011 – 2014			
		# of cases detected by SS		# of cases captured under HMIS	
		N	%	N	%
1	East Champaran	742	24.8	725	97.7

2	Muzaffarpur	1042	34.8	1008	96.7
3	Samastipur	445	14.9	429	96.4
4	Saran	767	25.6	688	89.7
Total		2996	100.0	2850	95.1

Discussion

Government of India has taken all possible efforts to eliminate kala-azar by the year 2020 but still the incidence of the diseases continues to be high in 64 affected blocks in the country, and even the disease is spreading in newer areas. Good quality Kala-azar surveillance in India always poses challenge to the programme. For effective control of VL disease within the targeted time period, a proper documentation of the extent of problem in terms of Kala-azar, PKDL, co-infections badly required. Proper monitoring of kala-azar elimination goals (<1 case/ 10 000 population) and deaths are warranted. The heath facility-based sentinel site surveillance may be useful to observe trends of kala-azar morbidity. Further, it may also be helpful in improving case management. The basic purpose of this system was to detect VL, PKDL outbreaks through regular analysis and reporting to plan appropriate public health responses. It is always challenging to capture complete and accurate patient level data in the existing surveillance system. The use of diagnostic testing may improve patient care and reduce the uses of unnecessary anti KA drugs. The sentinel site surveillance may provide the information on treatment outcomes. This approach was attempted first time in Bihar under research mode with fewer additional resources by including a few facilities at different levels to provide reliable and

complete information on kala-azar, PKDL and co-infection. We found that throughout four years of sentinel surveillance, kala-azar was an important contributor to both inpatient and outpatient.

The overall proportion of kala-azar cases were 14.29%, amongst the symptomatic cases (20968; fever more than 15 days) attended sentinel sites. This information is not available for routine existing surveillance system (Program data). However, a study conducted in India Nepal and Bangladesh reported proportion of kala-azar cases as 2.6% amongst symptomatic in India[14]. The proportion of new and old cases (unresponsive and relapse cases) were 86.1% and 13.9% respectively. Thus, 13.9% old VL cases were also diagnosed and treated at these sentinel sites. On the other hand, generally treatment of unresponsive and relapse cases were not done at the existing health facilities under the program. Such cases mostly refer to higher health facilities in the existing health system. Overall, decreasing trends of incidence per 10 000 population was noticed from 2011 to 2014 for all sites. Kala-azar was significantly reduced by more than 50% from 4.13/10 000 to 1.75/10 000 with mean 2.61 and 95% CI (2.26-3.41) for the study period. In 2014, about (38%) sites reached to elimination target (<1case per 10000 populations). The results are identical as described in Accelerated Plan for Kala-azar Elimination, 2017[15]. Suman Rijal et al. documented that, kala-azar cases has declined consistently from over 77 000 in the year1992 to less than 7000 cases in the year 2016. About, 8% of endemic units were above the threshold at the end of 2017[16]. Another study reported that VL incidence (cases/10,000/year) was reduced from 12.3 in the year 2007) to 0.9 in the year 2015. This is just below the target of World Health Organization’s threshold as a public health problem[17]. Our study revealed that, there was no uniform pattern of trends for medical college level sites. The possible explanation for the trend obtained for medical college level may be, training, frequent stakeholders meeting,

availability of free diagnostics and single dose AmBisome treatment and payment of various incentives under programme.

Monthly distributions of disease showed higher (11.2%, 11.7%, 10.0% and 8.5%) incidences for the months of January, March, July and August respectively for every year. However, it was lesser (5.8%, 6.1%, 6.7%) for the month of April, September and November. A study conducted by Paritosh Malviya et al. also reported highest peak in March–April and another one in July[18]. Whereas a study conducted in Sri Lanka reported seasonal trend of leishmaniasis incidence with a peak in July to September between the two monsoon periods and a drop in the number of reported cases compared to rest of the months[19].

The number of PKDL cases was almost double in Muzaffarpur and Samastipur district in the year 2013 when compared to 2011. On the whole co-infection was also found almost double (29) in 2014 when compared to 2011 (16), which showed rising trends for cases of co-infections too. In East Champaran district, co-infection was found very alarming i.e. more than 200% times increased in number of co-infection cases from 2011 to 2014. The overall proportion of co-infection and PKDL were 2.80% and 1.74% respectively. Finding may be vital for programme perspective and stakeholders. It was observed that, the incidence of kala-azar has decreased while incidence of PKDL and co-infections has increased in a span of four years of the study.

Das et al. documented that, the prevalence of PKDL was high (9.7 per 10 000)[20]. However, Singh RP et al. reported the prevalence of confirmed PKDL as 4.4 per 10 000 individuals[21]. On the other hand, V Ramesh et al. found an upward trend in reporting of PKDL cases to Safdarjung Hospital, New Delhi, India since the year 2005[22]. The demographic distribution of overall illness revealed that, overall VL+co-infection were slightly higher (55.6%) for males as compared to females (44.4%). Sakib Burza et al. reported the Relative Risk (RR) of being HIV-positive was

3.7 times higher for males[23]. Similarly, proportion of PKDL was also higher (52.8%) for males as compared to females (47.2%). This finding was identical with that of a study conducted by V Ramesh et al.[22] The prevalence of co-infection was recorded highest (3.34%) for the age-group 16-44 years as reported by Sakib Burza et al.[23] Majority of the cases (87%) were from rural areas. The possible explanations may be prevailing situation of the rural areas viz. favorable environmental conditions for vector, poor level of literacy, lack of awareness, low socio-economic conditions, poor living standard, and vegetation around houses and presence of cattle-shed inside houses as compared to urban areas. The proportion of HIV-VL co-infection was significantly higher (1.6%; $p<0.05$) as compared to other co-infection. This result is in line with other earlier studies conducted elsewherec[24-26]. There was significant weight loss in the kala-azar patients who have fever of longer duration (> 30 days). Significantly higher proportion (61%) of kala-azar cases was with 2-3 cm spleen size at the time of diagnosis. Similar findings were obtained by earlier studies conducted elsewhere [27-32]. Amongst the reproductive age-group patients (625), 3.7% (23) were found pregnant. None of the pregnant woman encountered with teratogenic effect in the delivered child. Identical result was obtained by a study conducted in Bihar by Pandey K et al.[33] Maximum cases (47%) were in the age group of 16 – 44 years and proportions of male in all age groups were higher and found statistically significant (Chi-square test = 7.6; $P<0.026$). These findings were coinciding with other earlier conducted studies[34&35]. Our results further showed that, significantly higher proportion (69%) of kala-azar cases were from up to middle level of education, and out of that, 33% were illiterate ones. The educational status between male and female categories were also found statistically significant (Wilcoxon test; <0.007). About, 48% of Kala-azar cases were from low socio-economic (agricultural laborer) groups. The male and female categories among the different occupational groups

showed statistically significant (*P<0.0001*) result. Significantly higher proportion (51%) of kala-azar cases was from schedule caste categories, which was similar with that of other studies conducted in other parts of world[36&37].

Diagnostics analysis

The overall, percentage of rK-39 positivity was about 14%, consistent in males and females. The sensitivity of splenic aspirate was found significantly higher (94.3%), followed by bone marrow (80.1%). However, the sensitivity of slit-skin smear/ skin-biopsy was found 55.2%. As regard to co-infection, maximum sensitivity was recorded as 42.7% for ELISA (HIV). These results were coinciding with that of other studies conducted in different parts of world[38-40].

Efficacy of anti kala-azar drugs administered at sentinel sites

Statistically significant differences in efficacies were observed for SAG (t-test = 6.98; *P<0.0009*), SAG + Miltefosine (t-test = 7.49; *P<0.0007*), Miltefosine (t-test = 5.57; *P<0.002*), Amphotericin B (t-test = 4.46; *P<0.002*), Miltefosine + Paramomycin (t-test = 7.93; *P<0.03*) and Ambisome (t-test = 2.06; *P<0.09*). The initial and final cure rate of Ambisome was found excellent (100%). Possible explanation may be the impact of elimination initiative (enhance awareness among people) highest proportion comes to the sites by own. The sensitivity of SAG was found very poor. Findings were identical with other study conducted by Om Prakash Singh et al in Bihar, India[41]. The relapse rate of SAG was found significantly higher as compared to other drugs (95%CI 0.2165 to 19.7035; *P<0.03*). Interestingly, no relapse was observed for Ambisome and Miltefosine + Paramomycin, however, it was negligible (<1%) for Miltefosine alone and Amphotericin B. These were in the line of findings of other studies conducted in Indian sub-continent[42-44]. There was not a single case of LAMA/defaulter with Ambisome and Miltefosine + Paramomycin. However, no significant differences of death rate were observed

between the different anti-kala-azar drugs (*P>0.06*). These findings were similar as reported by Sakib Burza et al[44].

Focused villages of sentinel sites having > 10 cases/year during the study period

Out of the total cases registered (2996) at sentinel sites, 10% share were from only 6 villages (304 cases) under two sentinel sites Kalyanpur and Madhuban of East Champaran district. The highest cases (19) were recorded in Shambu Chak village under Kalyanpur sentinel site of East Champaran district in each year. These villages are crucial for the program manager and policy makers and focused intervention need to be initiated on urgent basis to meet the target of kala-azar elimination. The possible explanations may be the continued transmission of the disease due to favorable conditions to sand fly in these pockets and lacking of various program interventions. Villages having more than 10 kala-azar cases per year required immediate specific intervention to control the disease and to check the transmission cycle. A researcher, Canjun Zheng et al. found similar results. He has concluded that VL continues to be a serious public health problem in Kashi Prefecture, China. It is a relatively high-risk area and there are several hot spots. The detection of spatial and spatiotemporal patterns can provide significant information for prevention and control measures[45]. However, another study conducted in India recorded the spatially clustered patterns with significant differences by village. The hotspots showed the spatial trend of kala-azar diffusion ($P < 0.01$)[46].

Concordance between HMIS and Sentinel sites data

Analysis showed that significantly higher proportion (95%) of cases was captured under HMIS. HMIS data almost close to actual disease burden. The similar pattern was noted for every district except Saran district (89.7%). This finding was not coinciding with that of other study (2010) conducted much earlier in India. This study reported the overall magnitude of VL cases not

reported to the government agencies was higher by a factor 4.17 (95% CI = 3.75–4.63)[47]. The possible explanations may be the various enforced interventions rooted under kala-azar elimination programme in endemic districts by the National Program. Niyamat Ali Siddiqui et al documented that a small proportion (38; 15.8%) of KA cases was not present in the public health system record. This was similar to present study finding[48].

Conclusions

This paper discusses various difficulties coming into programme implementation. It is focusing on existing kala-azar HMIS performance: 1) timely and accurate data reporting process; 2) accounting extent of problem in terms of kala-azar, PKDL, co-infections; 3) opportunity to improve kala-azar case management; 4) to identify outbreaks and hot spot; 5) capturing complete and accurate patient-level data; and 6) monitoring progress towards the national Kala-azar elimination goals.

Present study showed significant improvements in kala-azar, PKDL and co-infection case management at the sites. It has impacted on the proportion of patients with suspected kala-azar referred for diagnostic testing at elsewhere. At the onset of the sentinel site, majority of suspected kala-azar cases were diagnosed and treated at the facility itself without further referral to elsewhere. Utilization of laboratory services were much improved at the study sites. The majority (98%) of patients with suspected VL; PKDL & Co-infections were managed in the sentinel sites itself. Treatment practices were also significantly improved as per the national guidelines. Several studies conducted in India reported delay in the diagnosis of suspected individuals even when these services are available. Data come out from this study may also be vital for informing routine program decision making. It may further be useful in monitoring commodity stocks and demands. The burden of kala-azar expected at public health facilities may

be estimated with the help of findings of this study. Program Managers may use results for addressing a variety of local needs on various aspects. This method may be utilized in evaluating program effectiveness.

The success of any program required patience, flexibility, feedback from heath care workers, and continuous support from the government and funding agency. The kala-azar surveillance program described here may not be expanded beyond the sentinel sites. However, the lessons learned from this program should benefit other initiatives aimed to improving Kala-azar case management in other health care facilities. It may be useful for changing the practices of health care workers in India. Such systems may be utilized for deciding operational strategies to locate foci of increased KA, PKDL, and co-infection transmission. It provides lead to how to achieve high quality surveillance data in a resource limited setting.

Ethics statement

This study was approved by the Institutional Ethics Committee of RMRIMS, Patna vide approval No.01/IEC/2014 dated 28th January, 2014. The study specific written informed consent was obtained from head of the households.

Acknowledgements:

We are thankful to the Chief Medical Officer of Saran, Medical Officer In-charge of concerned PHCs for extending their support and co-operation for smooth execution of this study. Technical services rendered by Mr. Santosh Kumar Sinha, Technician, Department of Immunology, Mr. Madan Mohan Azad, Data Manager, Clinical Medicine is highly acknowledged and appreciated.

Financial support: Extramural (WB/NVBDCP)

References

1. Vidya Nand Ravi Das, Ravindra Nath Pandey, Niyamat Ali Siddiqui, Lloyd A.C. Chapman, Vijay Kumar, Krishna Pandey, Greg Matlashewski, Pradeep Das Longitudinal Study of Transmission in Households with Visceral Leishmaniasis, Asymptomatic Infections and PKDL in Highly Endemic Villages in Bihar, India. PLoS Negl Trop Dis 10(12): e0005196. doi:10.1371/journal.pntd.0005196.

2. Singh VP, Ranjan A, Topno RK, et al. Short Report: Estimation of underreporting of visceral leishmaniasis cases in Bihar, India. *American Journal of Tropical Medicine and Hygiene* 2010, 82(1): 9-11.

3. Singh SP, Reddy DCS, Rai M, Sundar S. Serious underreporting of visceral Leishmaniasis through passive case reporting in Bihar, India. *Tropical Medicine and International Health* 2006, *11*: 899-905.

4. Das P, Samuels S, Desjeux P, et al. Annual incidence of visceral leishmaniasis in an endemic area of Bihar, India. *Tropical Medicine and International Health* 2010, *15 (2)*: 4-11.

5. Salotra P, Singh R, et al. (2006) Challenges in the diagnosis of post kala-azar dermal leishmaniasis. Indian Journal of Medical Research 123: 295.

6. World Health Organization: Regional Strategic Framework for Elimination of Kala-azar from the South-East Asia Region (2011±2015), New Delhi: Regional Office for South-East Asia, 2014.

7. Breman JG, Holloway CN (2007) Malaria surveillance counts. Am J Trop Med Hyg 77: 36–47.

8. Bryce J, Roungou JB, Nguyen-Dinh P, Naimoli JF, Breman JG (1994) Evaluation of national malaria control programmes in Africa. Bull World Health Organ 72: 371–381.

9. Gething PW, Noor AM, Gikandi PW, Ogara EAA, Hay SI, Nixon MS, Snow RW, Atkinson PM: Improving imperfect data from health management information systems in Africa using space-time geostatistics. PLoS Med 2006, 3:e271.

10. Health Metrics Network: Country Health Information Systems Assessments: overview and lessons learned. In Health Merics Network Network, Editor. Geneva: World Health Organization; 2012.

11. Joshua O Yukich, Jessica Butts, Melody Miles, Yemane Berhane, Honelgn Nahusenay, Joseph L Malone, Gunawardena Dissanayake, Richard Reithinger and Joseph Keating. A description of malaria sentinels surveillance: a case study in Oromia Regional State, Ethiopia. Malaria Journal 2014, 13:88.

12. Asadu Sserwanga, Jamal C. Harris, Ruth Kigozi, Manoj Menon, Hasifa Bukirwa, Anne Gasasira, Stella Kakeeto, Fred Kizito, Ebony Quinto, Denis Rubahika, Sussann Nasr, Scott Filler, Moses R. Kamya, Grant Dorsey. Improved Malaria Case Management through the Implementation of a Health Facility-Based Sentinel Site Surveillance System in Uganda. PLoS ONE; January 2011 | Volume 6 | Issue 1 | e16316.

13. S.K. Jain, J.K. Aggarwal, S. Rajpal and U. Baveja. Prevalence of HIV infection among tuberculosis patients in Delhi – A sentinel surveillance study. Ind. J. Tub., 2000, 47,21.

14. Dinesh Mondal, Shri Prakash Singh, Narendra Kumar, Anand Joshi, Shyam Sundar, Pradeep Das, Hirve Siddhivinayak, Axel Kroeger, Marleen Boelaert. Visceral Leishmaniasis Elimination Programme in India, Bangladesh, and Nepal: Reshaping the

Case Finding/ Case Management Strategy. PLOS NTD; January 2009 | Volume 3 | Issue 1 | e355.

15. Accelerated Plan For Kala-azar Elimination, 2017, Directorate, National Vector Borne Diseases Control Programme, 2017.

16. Suman Rijal and colleagues. Eliminating visceral leishmaniasis in South Asia: the road ahead. BMJ 2019; 364:k5224 | doi: 10.1136/bmj.k5224 Page, 1-5.

17. Caroline A. Bulstra, Epke A. Le Rutte, Paritosh Malaviya, Epco C. Hasker, Luc E. Coffeng, Albert Picado, Om Prakash Singh, Marleen C. Boelaert, Sake J. de Vlas and Shyam Sundar. Visceral leishmaniasis: Spatiotemporal heterogeneity and drivers underlying the hotspots in Muzaffarpur, Bihar, India PLOS Neglected Tropical Diseases 2018; Page 1-21. https://doi.org/10.1371/journal.pntd.0006888.

18. Paritosh Malaviya, Albert Picado, Shri Prakash Singh, Epco Hasker, Rudra Pratap Singh, Marleen Boelaert, Shyam Sundar. Visceral Leishmaniasis in Muzaffarpur District, Bihar, India from 1990 to 2008. PLoS ONE 2011,Volume 6; Issue 3; e 14751, Page 1-7. www.plosone.org.

19. Lahiru Sandaruwan Galgamuwa, Samath D. Dharmaratne and Devika Iddawela Leishmaniasis in Sri Lanka: spatial distribution and seasonal variations from 2009 to 2016. Parasites & Vectors 2018, 11:60. DOI 10.1186/s13071-018-2647-5.

20. Vidya N R Das, Niyamat A Siddiqui, Krishna Pandey, Chandra S Lal, Sanjay K Sinha,Sanjiva Bimal, Roshan K Topno, Shubhankar K Singh, Sunil Kumar, Pradeep Das. The usefulness of trained field workers in diagnosis of post-kala-azar dermal leishmaniasis (PKDL) and clinico-epidemiological profile in highly endemic areas of Bihar.

Transactions of The Royal Society of Tropical Medicine and Hygiene, Volume 113, Issue 6, June 2019, Pages 332–340, https://doi.org/10.1093/trstmh/trz007.

21. Singh RP, Picado A, Alam S, Hasker E, Singh SP, Ostyn B, Chappuis F, Sundar S, Boelaert M. Post-kala-azar dermal leishmaniasis (PKDL) in visceral leishmaniasis-endemic communities in Bihar, India. Tropical Medicine & International Health: TM & IH [28 Dec 2012] DOI: 10.1111/tmi.12044.

22. V. Ramesh, Himanshu Kaushal, Ashwani Kumar Mishra, Ruchi Singh and Poonam Salotra. Clinico-epidemiological analysis of Post kala-azar dermal leishmaniasis (PKDL) cases in India over last two decades: a hospital based retrospective study. BMC Public Health (2015) 15:1092. DOI 10.1186/s12889-015-2424-8.

23. Sakib Burza, Raman Mahajan, Prabhat K. Sinha, Johan van Griensven, Krishna Pandey, Marı´a Angeles Lima, Marta Gonzalez Sanz, Temmy Sunyoto, Sunil Kumar, Gaurab Mitra, Ranjeet Kumar, Neena Verma, Pradeep Das. Visceral Leishmaniasis and HIV Co-infection in Bihar, India: Long-term Effectiveness and Treatment Outcomes with Liposomal Amphotericin B (AmBisome) PLOS Neglected Tropical Diseases | www.plosntds.org 1 August 2014 | Volume 8 | Issue 8 | e3053.

24. Rahman MF, Banu S, Alam MJ, Uddin MJ, Sarkar MAS, Basher A, Nath P, Hakim M, Sanjoba C, Paul S, Alim MA, Matsumoto Y, Rahman MB. Leishmaniasis amongst TB patients from Kala-azar endemic areas admitted to Surya Kanto Hospital, Mymensingh JAFMC Bangladesh. Vol 12, No 2 (December) 2016 Page 3-8.

25. Van Griensven J. Visceral leishmaniasis and HIV coinfection in Bihar, India: a wake-up call? Clin Infect Dis 2014; 59:556–8.

26. Sakib Burza, Raman Mahajan, Marta Gonzalez Sanz,Temmy Sunyoto, Ranjeet Kumar, Gaurab Mitra, and María Angeles Lima. HIV and Visceral Leishmaniasis Coinfection in Bihar, India: An Under recognized and Under diagnosed Threat Against Elimination BRIEF REPORT • CID 2014:59 (15 August); Page:552-555.

27. Abdelsalam M. Nail, Abdelmageed M. Imam. Visceral leishmaniasis: Clinical and demographic features in an African population. Pak J Med Sci 2013; 29(2): 485-489. doi: http://dx.doi.org/10.12669/pjms.292.3151.

28. Agrawal Y, Sinha AK, Upadhyaya P, Kafle SU, Rijal S, Khanal B. Hematological profile in visceral leishmaniasis. Int J Infect Microbiol 2013; 2(2):39-44. DOI: http://dx.doi.org/10.3126/ijim.v2i2.8320.

29. Aritra Das, Morchan Karthick, Shweta Dwivedi, Indranath Banerjee, Tanmay Mahapatra , Sridhar Srikantiah, Indrajit Chaudhuri. Epidemiologic Correlates of Mortality among Symptomatic Visceral Leishmaniasis Cases: Findings from Situation Assessment in High Endemic Foci in India. PLOS Neglected Tropical Diseases | DOI:10.1371/journal.pntd.0005150 November 21, 2016 1 / 12.

30. Mittal V, Bhatia R, Sehgal S. Clinico-epidemiological profile of kala-azar patients in Delhi. J Commun Dis. 1989 Dec; 21(4):255-61.

31. Sarker CB, Chowdhury KS, Siddiqui NI, Jamal MF, Rahman S, Momen A, Dhar DK, Alam KS. Clinical profile of Kala-azar in adults: as seen in Mymensingh Medical College Hospital, Mymensingh, Bangladesh. Mymensingh Med J. 2003 Jan; 12(1):41-4.

32. Kumar Saurabh, Shilpi Ranjan, Rajeev Ranjan Prasad. Clinical and haematological parameters associated with patients of visceral leishmaniasis in a district of North Bihar

International Journal of Community Medicine and Public Health. Vol 4, No 6 (2017). DOI: http://dx.doi.org/10.18203/2394-6040.ijcmph20172156.

33. Krishna Pandey, Vidyanand Ravidas, Niyamat A. Siddiqui, Sanjay K. Sinha, Rakesh B. Verma, Tripurari P. Singh, A. C. Dhariwal, R. K. Das Gupta, and Pradeep Das. Pharmacovigilance of Miltefosine in Treatment of Visceral Leishmaniasis in Endemic Areas of Bihar, India Am J Trop Med Hyg. 2016 Nov 2; 95(5): 1100–1105. doi: 10.4269/ajtmh.16-0242.

34. Epco Hasker, Shri Prakash Singh, Paritosh Malaviya, Albert Picado, Kamlesh Gidwani, Rudra Pratap Singh, Joris Menten, Marleen Boelaert, and Shyam Sundar. Visceral Leishmaniasis in Rural Bihar, India. Emerging Infectious Diseases. www.cdc.gov/eid Vol. 18, No. 10, October 2012 DOI: http://dx.doi.org/10.3201/eid1810.111083;1662-1664.

35. Agrawal Y, Sinha AK, Upadhyaya P, Kafle SU, Rijal S, Khanal B. Hematological profile in visceral leishmaniasis. Int J Infect Microbiol 2013; 2(2):39-44 DOI: http://dx.doi.org/10.3126/ijim.v2i2.8320:39-44.

36. Adam Suleiman Abake, Altagi Abdalla Mohammed, Abd Elbasit Elawad. Socioeconomic and behavioural risk factors for infection of visceral leishmaniasis Gedaref state – Sudan 2015. Global Journal of Medicine and Public Health. www.gjmedph.com Vol. 6, No. 2 2017; 1-7.

37. Abdelsalam M. Nail, Abdelmageed M. Imam.Visceral leishmaniasis: Clinical and demographic features in an African population Pak J Med Sci 2013 Vol. 29 No. 2 www.pjms.com.pk 485; 485-489 doi: http://dx.doi.org/10.12669/pjms.292.3151.

38. Yazezew Kebede Kiros and Bethlhem Feleke Regassa. The role of rk39 serologic test in the diagnosis of visceral leishmaniasis in a Tertiary Hospital, Northern Ethiopia. Kiros and Regassa BMC Res Notes (2017) 10:169 DOI 10.1186/s13104-017-2490-3:1-5.

39. Mauro RobertoB, DA Silva, Jay M. Stewart, and Carlos H. Costa. Sensitivity of bone marrow aspirates in the diagnosis of visceral leishmaniasis. J. Trop. Med. Hyg., 72(6), 2005, pp. 811–814.

40. Sarker CB, Alam KS, Jamal MF, Rahman S, Huq MH, Musa AK, Sutradhar SR, Talukder SI, Debnath CR. Sensitivity of splenic and bone marrow aspirate study for diagnosis of kala-azar. Mymensingh Med J. 2004 Jul; 13(2):130-3.

41. Om Prakash Singh, Bhawana Singh, Jaya Chakravarty and Shyam Sundar. Current challenges in treatment options for visceral leishmaniasis in India: a public health perspective. Infectious Diseases of Poverty (2016) 5:19. DOI 10.1186/s40249-016-0112-2.

42. Vishal Goyal, Sakib Burza, Krishna Pandey, Shambhu Nath Singh, Ravi Shankar Singh, Nathalie Strub-Wourgaft , Vidya Nand Rabi Das , Caryn Bern, Allen Hightower, Suman Rijal, Temmy Sunyoto, Fabiana Alves, Nines Lima, Pradeep Das and Jorge Alvar. Field effectiveness of new visceral leishmaniasis regimens after 1 year following treatment within public health facilities in Bihar, India. PLOS Neglected Tropical Diseases | https://doi.org/10.1371/journal.pntd.0007726 September 26, 2019 2 /10.

43. Suman Rijal, Bart Ostyn, Surendra Uranw, Keshav Rai, Narayan Raj Bhattarai, Thomas P. C. Dorlo, Jos H. Beijnen, Manu Vanaerschot, Saskia Decuypere, Subodh S. Dhakal, Murari Lal Das, Prahlad Karki, Rupa Singh, Marleen Boelaert, and Jean-Claude Dujardin. Increasing Failure of Miltefosine in the Treatment of Kala-azar in Nepal and the Potential Role of Parasite Drug Resistance, re-infection, or Noncompliance CID 2013:56 (1 June).

Clinical Infectious Diseases, Volume 56, Issue 11, 1 June 2013, Pages 1530–1538, https://doi.org/10.1093/cid/cit102.

44. Sakib Burza, Prabhat K. Sinha, Raman Mahajan, María Angeles Lima, Gaurab Mitra, Neena Verma, Manica Balasegarem and Pradeep Das. Five-Year Field Results and Long-Term Effectiveness of 20 mg/kg Liposomal Amphotericin B (Ambisome) for Visceral Leishmaniasis in Bihar, India. PLOS Neglected Tropical Diseases | www.plosntds.org 1 January 2014 | Volume 8 | Issue 1 | e2603.

45. Canjun Zheng, Jingying Fu, Zeng Li, Gang Lin, Dong Jiang and Xiao-nong Zhou. Spatiotemporal Variation and Hot Spot Detection of Visceral Leishmaniasis Disease in Kashi Prefecture, China. International Journal of Environmental Research and Public Health. 2018, 15, 2784; doi: 10.3390/ijerph15122784 www.mdpi.com/journal/ijerph.

46. Gouri Sankar Bhunia, Shreekant Kesari, Nandini Chatterjee, Vijay Kumar and Pradeep Das. Spatial and temporal variation and hotspot detection of kala-azar disease in Vaishali district (Bihar), India. BMC Infectious Diseases 2013, 13:64 http://www.biomedcentral.com/1471-2334/13/64.

47. Vijay P. Singh, Alok Ranjan, Roshan K. Topno, Rakesh B. Verma, Niyamat A. Siddiqui, Vidya N. Ravidas, Narendra Kumar, Krishna Pandey, and Pradeep Das. Short Report: Estimation of Under-Reporting of Visceral Leishmaniasis Cases in Bihar, India. Am. J. Trop. Med. Hyg., 82(1), 2010, pp. 9–11 doi:10.4269/ajtmh.2010.09-0235.

48. NA Siddiqui, K Pandey, VNR Das, SK Sinha, RB Verma, CS Lal, V Ali, RK Topno, MR Dikhit and P Das. A preliminary study assessing magnitude of unreported kala-azar cases in a highly endemic district of Bihar, India: A positive impact of Indian elimination

programme. 2020; Journal of Vector Borne Diseases (Reference JVBD_113_18 Accepted for publication in December issue, 2020).
