# Supplementary material for: Improved kala-azar case management through implementation of health facility-based sentinel sites surveillance system in Bihar, India
Source: PLoS Negl Trop Dis. 2021 Aug 24;15(8):e0009598. doi: 10.1371/journal.pntd.0009598 (PMC8384155; doi:10.1371/journal.pntd.0009598)
Supplement: Attachment — Submitted filename: Reply to the reviewers comments.docx [file pntd.0009598.s002.docx]

**REPLY TO REVIEWER’S COMMENTS**

Reviewer #1:

**Query # 1**

The description of the materials and methods are very explorative. Some of these have little scientific value. It would be advisable to publish the protocols on a public site and refer to this site in this manuscript. By doing so, the useful information will be more accessible by non-scientific members (i.e. decision makers)

**Answer**

Suggestions of learned reviewer has been accepted and accordingly modified (shorten) the description of the materials and methods in the lines no-298-303 at page no-16, 319-334 at page no-17, 347-354 at page no-18, 366-367, 372-379 at page no-19, 412-418, 422-425 at page no-21 and line no-426-433 at page no-22 respectively. As suggested, complete protocol containing detail description of material and method are being made available in the institute website [www.rmrim.org.in](http://www.rmrim.org.in) for non scientific member (i.e. Decision maker)

Reviewer #2:

**Query # 1**

Are the objectives of the study clearly articulated with a clear testable hypothesis stated?

Yes, partially. The objective of the efficacy of anti kala-azar drugs administered at sentinel sites was not stated among the objectives.

**Answer**

As suggested, it has been added in the lines no 161-162 at page no-8.

**Query # 2**

Is the study design appropriate to address the stated objectives?

Yes, except for the efficacy of anti kala-azar drugs

**Answer**

As suggested by the learned reviewer, responses to different anti-kala-azar drugs administered at sentinel sites have been added in place of efficacy of anti-kala-azar drugs. It has been addressed in line no-571, 576 at page no-34, 580, 583, 586 & 589 at page no-35 and in line no-769, 774 at page no-45. Further, it has been addressed in line no-785 at page no-46

**Query # 3**

Is the population clearly described and appropriate for the hypothesis being tested?

Yes.

**Answer**

No response is required

**Query # 4**

Is the sample size sufficient to ensure adequate power to address the hypothesis being tested?

Yes.

**Answer**

No response is required

**Query # 5**

Were correct statistical analysis used to support conclusions?

Yes, except for the efficacy of anti kala-azar drugs.

**Answer**

As suggested by the learned reviewer, efficacy of anti kala-azar drug has been corrected with response to different anti-kala-azar drugs administered at sentinel sites in lines no-571, 576 at page no-34, 580, 583, 586 & 589 at page no-35 and line no-769, 774 at page no-45. Further, it has been addressed in line no-785 at page no-46.

**Query # 6**

Are there concerns about ethical or regulatory requirements being met?

Yes.

**Answer**

No response is required

Reviewer #3:

The Manuscript "Improved Kala-azar Case Management through Implementation 1 of Health Facility-Based Sentinel Sites Surveillance System in Bihar, India" is a well planned and well implemented work related to a very important public health problem in India. The work describes kala-azar, a neglected vector-borne disease, targeted for elimination, and becomes impeded by high incidence of cases in several affected rural blocks of Bihar and the spread of disease in newer areas. The present study proposed to do a proper accounting of VL, PKDL and co-infection morbidity, mortality, diagnosis, case management, hotspot identification and monitoring the impact of elimination interventions.

The manuscript warrants publication. The study hypothesis and the design are sound using proper statistical methods.

I reckon the authors have met the ethical and regulatory requirements.

**Answer**

No response is needed

Reviewer #1:

The results section is very well elaborated.

However some attention points have to be addressed:

**Query # 1**

A lot of data are presented but are not discussed. By doing so, the reader is left behind to self explore the added value of all these results.

**Answer**

Agreed and as per the suggestion of reviewer, remaining important results have been discussed in the discussion section and added in line no-724-728, 730-731 at page no-43. Further, it has been added in the line no-746-748, 750-752, and 755-756 at page no-44. Further, added in line no-893-895 at page no-50, 896-903 and in line no-908-916 at page no-51.

**Query # 2**

Figure 2: the values added in the figure for the co-infection (red line) does not correspond with the scale of the incidence in the coordinate. For instance the point for 2011 (0.06) is in the figure >0.08, the point for 2014 (0.1) is in the figure >0.16. Therefor, the red line should be lower in the figure. Alse the values of 2011 and 2012 are both 0.06, however in the figure they does not seem at equal level.

**Answer**

Agreed and replaced the Figure-2 with new Graph-1 at page no-27.

**Query # 3**

Table 3: the “%” deserves more explanation. If I understood, it means the percentage of positive tests on the amount of test performed for the specific disease (not for all tests performed).

- Also for this table, describe the abbreviations used.

- Related to this, the % used in the section “Diagnostics analysis” in the discussion is very confusing for the reader. It should be described what is meant by the %.

**Answer**

Agreed with the suggestion of learned reviewer, accordingly explanation for percentage used in the table has been provided in the modified table. As suggested, abbreviations are described below modified table. Modified table has been presented at page no-34. Further, % used under sub heading “Diagnostics analysis” has been clearly described (meaning of %) in the discussion section in the line no-758-761 at page no-44 and in line no-762-764 at page no-45.

**Query # 4**

Table 5: It is not clear for the reviewer how the % is calculated. Please describe. Related to this, the reviewer could not interpret this part of the result section.

**Answer**

Table-5 has been modified at page no-38 & 39. The description of % have also been provided in the table and text in line no-625-627 at page no-38 in results section and in line no-826-827 at page no-47 in discussion section.

Improvements to make to some difficult to understand sentences, repetitions, lack of additional data:

**Query # 5**

L432-434: "A statistical significant difference was found between villages having 1-2 cases (74%) and villages with 3-5 cases (15%). Good proportion (4%) of villages was still with more than 10 cases."

**Answer**

As suggested, with the help of additional data sentences have been modified from line no-466-469 at page no-23.

**Query # 6**

L439: "About half of the VL cases (47%) were in the age-group 16-44 years." This should be compared with the description of the population. What is the fraction of the 16-44 years group within the entire population?

**Answer**

As suggested, sentence has been modified from line no-476-478 at page no-24.

**Query # 7**

L445: "Overall, mean value of the incidence was recorded 2.61 with 95% CI as 2.26 to 3.41.”This is a repetition of line L441 and can thus be omitted.

**Answer**

Agreed and omitted in the line no-486 at page no-24.

Reviewer #2:

**Query # 1**

Does the analysis presented match the analysis plan?

Yes.

Are the results clearly and completely presented?

Yes, however there are a lot of abbreviations stated for the first time without full name which made it difficult to follow the text smoothly. I also suggest the authors to provide a table for abbreviations that have been used over the manuscript.

**Answer**

Agreed and added a table for abbreviations at page no-53-54, which can be used over the manuscript.

Reviewer #3:

**Query # 1**

There come some clarifications to be made

i) The sentence "20968 symptomatic cases attended sentinel sites, 2996 cases of KA and 53 cases of PKDL were registered from 889 endemic villages" ; The proportion of new and old cases was 86.1% and 13.9% respectively. Out of 20,968 cases 2996 cases of KA and 53 cases of PKDL were registered. Where did the rest of the symptomatic go or attended to?

**Answer**

Out of 20968 (symptomatic cases; fever more than 15 days), only 3049 were diagnosed as KA and PKDL and rest were negative by rk-39 and as well as bone marrow/spleenic aspiration (KA)/slit skin smear examination (PKDL). These cases were referred to other departments (Malaria, TB, Filaria, leprosy, viral disease, etc) of the health facilities where sentinel sites were functioning for further management of their illness. It has been added in the lines no-461-464 at page no-23.

**Query # 2**

ii) A note on disparity in Government Vs, Private hospital based reporting system needs to be explained. Also, the observation of high prevalence of cases in some Castes. Is socioeconomic condition and/or genetic basis implicated?

**Answer**

Under sub heading “Concordance between KMIS and sentinel sites data” within the results section in line no-625-626 at page no-38, it has been documented that out of 2996 cases, about 95% (2850) cases were captured under current district KMIS database and not of Government Vs, Private hospital based reporting system. In spite of reporting of cases from sentinel sites, district KMIS database is missing on average 5% cases, which warrants that still efforts required strengthening current kala-azar Management Information System (KMIS).

However, under sub heading “Concordance between KMIS and sentinel sites data within the discussion section, a discussion was made about efficiency of Government surveillance system in capturing kala-azar cases and not of Government Vs, Private hospital based reporting system from lines no-826-827 at page no-47. It has been said that higher proportion (95%) of cases were captured under current district KMIS database. Further, it can be said that KMIS data is almost close to actual disease burden of the district. Whereas, earlier study conducted by Vijay P. Singh et al, 2010 reported the overall magnitude of VL cases not reported to the government surveillance system was higher by a factor 4.17 (95% CI = 3.75–4.63). A recent study conducted by N.A. Siddiqui et al, 2019 documented that only a small proportion (15%) of KA cases was not present in the government surveillance system. Studies discussed above are available in the discussion section from line no-869-872 at page no-49, and in line no-874-876 at page no-50. The possible explanations may be the various enforced interventions rooted under kala-azar elimination programme in endemic districts by the National Program. It has been added in line no-828-829 at page no-48

As per the suggestion, explanations have been provided in respect to Government Vs, Private hospital based reporting system. Accordingly previous Table-5 has been modified and presented at page no-38&39 in more understandable /meaningful manner.

Under sub heading “Demographic characteristics of the sentinel sites” within the results section from lines no 538-539, 542-543 at page no-29 and in discussion section from lines no-749-750, 753-754 at page no-44 revealed that about 51% of kala-azar cases were from schedule caste categories. The possible reasons for this may be mainly due to socio-economic conditions and not of due to genetic implications and it has been added in the line no-755-756 at page no-44. This explanation has been supported by other studies findings conducted in other parts of world discussed in lines no-754-755 at page no-44 in discussion section.

--------------------

<b>Conclusions</b></br></br>

-Are the conclusions supported by the data presented?</br>

-Are the limitations of analysis clearly described?</br>

-Do the authors discuss how these data can be helpful to advance our understanding of the topic under study?</br>

-Is public health relevance addressed?</br></br>

Reviewer #1:

**Query # 1**

The conclusion is supported by the data presented in the result section. The authors discuss the data in relation with other findings, available at international level.

**Answer**

No response is needed

Reviewer #2:

**Query # 1**

Are the conclusions supported by the data presented?

Partially

**Answer**

Agreed and additional results have been added in the conclusion section from line no-893-895 at page no-50, line no-896-898 at page no-51 and from line no-899-903, 908-916 at page no-51 to address the reviewer query.

**Query # 2**

Are the limitations of analysis clearly described?

Yes.

**Answer**

No response is needed

**Query # 3**

Do the authors discuss how these data can be helpful to advance our understanding of the topic under study?

Yes.

**Answer**

No response is needed

**Query # 4**

Is public health relevance addressed?

Yes.

**Answer**

No response is needed

Reviewer #3:

**Query # 1**

The sentence "However, generalizability of this sentinel surveillance finding to other context remains a major limitation of this study" needs clarification.

**Answer**

As suggested, clarifications are as under;

Nearly 80% of the total VL cases reported by the programme are from 12 districts of Bihar. Out of these 12 districts, sentinel sites were made in 4 highly endemic districts (Muzaffarpur, Samastipur, East Champaran and Saran) as per their Incidence proportion rate (IPR). In each district, two primary health centers (PHC’s) were selected as sentinel sites again as per the incidence proportion rate (Ranking 1 & 2). These districts and PHC’s were traditionally high endemic PHC’s since long back consistently. Additionally due consideration has been given to Program commitment for diagnostic (rk-39) and the first line anti kala-azar drug i.e. miltefosine throughout the study period in the sentinel sites. In addition, there were clause of fulfillment of readiness criteria at the each sentinel site (already described in the line no 171 to 180 at page no-8, 181-189 at page no-9 and 192-212 at page no-10. Rigorous efforts were taken to improve all the sentinel sites to meet the readiness criteria and research activities started only after meeting readiness criteria at the site. Therefore sentinel site surveillance described under the present study cannot be integrated to other set up (medium and low endemic areas). However, it can be integrated in highly endemic areas without program commitment and fulfillment of readiness criteria.

The required clarifications have been added from line no-67-76 at page no-4.

**Query # 2**

The manuscript highlights that the data coming out from this study may be vital for informing routine program decision making. It may further be useful in monitoring commodity stocks and demands. The burden of kala-azar expected at public health facilities may be estimated with the help of findings of this study. Program Managers may use results for addressing a variety of local needs on various aspects. This method may be utilized in evaluating program effectiveness.

**Answer**

No response is needed

--------------------

<b>Editorial and Data Presentation Modifications?</b></br><br/>

Use  this section for editorial suggestions as well as relatively minor modifications of existing data that would enhance clarity. If the only modifications needed are minor and/or editorial, you may wish to recommend “Minor Revision” or “Accept”.

Reviewer #1:

**Query # 1**

Multiple typing errors were encountered in this manuscript. It would be useful to recheck the document by a spelling checker or re-read the final document.

**Answer**

As suggested, rechecked the document with the help of spelling checker.

**Query # 2**

At different positions in the text, abbreviations are used without definition. It would be advisable to define the abbreviation when first used.

**Answer**

Agreed, and accordingly defined the abbreviation throughout the document.

**Query # 3**

See attachment for more details

**Answer**

Seen and corrected where required.

Reviewer #2: (No Response)

Reviewer #3:

**Query #**

Minor Revision that incorporates clarifications of some sentences is needed thane can be Accepted for Publication.

**Answer**

As suggested by the learned reviewer revision of sentences has been compiled.

--------------------

<b>Summary and General Comments</b></br></br>

Use this section to provide overall comments, discuss strengths/weaknesses of the study, novelty, significance, general execution and scholarship. You may also include additional comments for the author, including concerns about dual publication, research ethics, or publication ethics. If requesting major revision, please articulate the new experiments that are needed.

**Reviewer #1:**

This manuscript describes a global project for the neglected disease VL facing as well the surveillance, testing capacity, treatment and educational level of healthcare workers. It is an important study to help the decision makers to monitor the efficiency of control programs in the elimination of the KA. It would be as well important for other regions and countries to implement such a control program. It demonstrates the use of the effort made to improve the deficiencies and to harmonize the level of qualifications at the different sentinel sites.

**Query # 1**

It is however not clear which of these elements are prior in the reduction seen of the KA between 2011 to 2014.

**Answer**

The possible explanations for the reduction seen in the kala-azar cases between 2011 to 2014 are various programme interventions taken place at grass root level such as involvement of various stakeholders, frequent stakeholders meeting, and organization of frequent training programme for stakeholders as well as health system workers of the kala-azar control programme. The other interventions are capacity building of health system workers involved in kala-azar control programme, availability of free diagnostics, single dose AmBisome treatment, regular indoor residual spraying in endemic areas, payment of various incentives under programme, etc. In the discussion section, various studies are referred for declining trends of VL incidence (References; 15, 16 & 17). Few possible explanations for the decreasing trends of kala-azar disease are already available in line no-668-669 at page no-40 and 670-671 at page no-41.

**Reviewer #2:**

This study addressed an important neglected disease which is kala-azar. India, representing 50% of the global cases of the disease. The effort of the paper to explore possible interventions in terms of improving surveillance to identify kal-azar cases as early as possible and to treat them make the paper of reasonable importance. However, I have general and specific comments to the authors as followings: (The line number referred to here is the line number of the word file of the manuscript)

**Query # 1**

There have been a lot of abbreviations used through the manuscript, many of them used for the first time without full name. This would interrupt the focus of the reader. I would suggest you revise your abbreviations all over the paper and to write an additional table for your abbreviations so that the reader can check whenever needed.

**Answer**

Agreed and as suggested by the learned reviewer abbreviation has been revisited and provided throughout the document; as suggested an additional table for abbreviation has been added at page no-53-54, which can be used over the manuscript.

**Query # 2**

Abstract- you did not state clearly the objectives of the study.

**Answer**

The major objectives of the study were proper accounting of VL, PKDL and co-infection morbidity, mortality, diagnosis, case management, hotspot identification and monitoring the impact of elimination interventions. The other objectives were monitoring of real-time kala-azar and PKDL data for assessing changes in kala-azar/PKDL/Co-infection morbidity, mortality and pattern of disease transmission. In addition, responses to different anti kala-azar drugs administered at sentinel sites were also evaluated.

It is available in the line no-32-33 at page no-2 under subheading “background” of the Abstract. However as per the suggestion, major objectives have also been added under heading “Introduction” from line no-158-162 at page no-8.

**Query # 3**

Abstract line 23, HMIS abbreviation appears for the first time, and has to be written in full name.

**Answer**

It has been corrected in the line no-26 at page no-2.

**Query # 4**

Abstract, line 26, KA, this abbreviation appears for the first time, and has be written in full name.

**Answer**

Full abbreviation of KA has been provided in the line no-30 at page no-2.

**Query # 5**

Abstract-methodology part- line 34, you need to define what do you mean by symptomatic cases?

**Answer**

As suggested by reviewer, descriptions of sysmptomatic cases have been added from line no-40-41 at page no-2.

**Query # 6**

Abstract- methodology part Line 46:  SAG abbreviation appeared for the first time within the text and has to be written in full name.

**Answer**

Abbreviation of SAG has been provided in the line no-53 at page no-3.

**Query # 7**

Abstract-principal findings part- you mentioned private health providers has low number of cases notification, how could we improve the private provider surveillance?- I suggest you to discuss this in some relevant part of the text.

**Answer**

Not agreed with the reviewer comment, because our analysis showed that significantly higher proportion (95%) of cases was captured under KMIS. KMIS data almost close to actual disease burden. Therefore low number of cases notification by private health care providers may not be a major concern for the programme. It means that kala-azar control programme is doing well at the moment (under the era of kala-azar elimination). However, it can be improved at private health care provider’s level through frequent training/re-training for detection of VL/PKDL cases in the endemic areas, making diagnostic (rk-39) and anti KA drugs in the open market freely. Government should make provision of incentive for them. Such approach enhance the detection of hidden and under reported case of VL/PKDL.

As suggested by learned reviewer, discussion on low number of cases notification by private health care providers has been added from line no-877-883 at page no-50.

**Query # 8**

Introduction- Line 96, is it manifest or manifests? grammar issue

**Answer**

It has been corrected in line no-114 at page no-6.

**Query # 9**

Introduction- line 107- is it depend or depends? grammar issue.

**Answer**

It has been corrected in line no-126 at page no-6.

**Query # 10**

Introduction, line 111, I think you mean counties and not countries, please check.

**Answer**

No correction is required as countries are correct

(PKDL and co-infections reported from 36 endemic countries including Bihar, India).

**Query # 11**

Material and methods- Line 147 to 151, reference is needed for this information.

**Answer**

It has been added (kala-azar incidence data provided to State Programme Office, Bihar) in the line no-175 at page no-8.

**Query # 12**

Material and methods- Line 179- d is a typo.

**Answer**

It has been corrected in the line no-205 at page no-10.

**Query # 13**

Material and methods-line 243-246, you need a reference for the case definition of Kala-azar that you used.

**Answer**

As per suggestion of learned reviewer, it is being added in the line no-275-276 at page no-14 (Accelerated plan for kala-azar elimination, 2017, WHO/NVBDCP).

**Query # 14**

Material and methods- line 248-250; you need a reference for the case definition of Post Kala-azar Dermal Leishmaniasis (PKDL) that you used.

**Answer**

As per suggestion of learned reviewer, it is being added in the line no-280-281 at page no-14 (Accelerated plan for kala-azar elimination, 2017, WHO/NVBDCP).

**Query # 15**

Material and methods- Diagnosis of KA and PKDL, you need a reference to show that the tests suggested to use to identify patients with KA and PKDL are approved by national or international authorities such as WHO.

**Answer**

As per suggestion of learned reviewer, it is being added in the line no-296-297 at page no-16 (Accelerated plan for kala-azar elimination, 2017, WHO/NVBDCP).

**Query # 16**

Material and method- Line 277- is there any reference for the standard protocol of Kala-azar and PKDL that you used in your study?

**Answer**

Yes, we have used the descriptions made in “accelerated plan for kala-azar elimination, 2017, WHO/NVBDCP) as reference for the standard protocol of kala-azar and PKDL.

**Query # 17**

Result-Line 518-Efficacy of anti kala-azar drugs administered at sentinel sites. Efficacy of drugs usually studied through Randomized Control Trails (RCT), which is not the case of your description here. Therefore, I would rather suggest you to rename this section with other title such as Response to anti kala-azar drugs administered at sentinel sites. If you need to talk about the efficacy of your different protocols to treat kala-azar then you have to carry out RCT.

**Answer**

Agreed, and modified accordingly throughout the results and discussion section. As per the suggestion of the reviewer, word ‘efficacy’ has been replaced by ‘response’.

**Query # 18**

Result- Line 544- ADR abbreviation used for the first time and has to be written in full name.

**Answer**

As suggested, abbreviation of ADR has been added in the line no-798 at page no-46.

**Query # 19**

Discussion- Line 593, you need to put coma after India.

**Answer**

Agreed and added accordingly.

**Query # 20**

Discussion- Line 604, it should be have and not has, grammar issue.

**Answer**

Agreed and corrected in the line no-663 at page no-40.

Discussion- general comment that this part failed to explain possible explanations of the majority of the results stated by the authors as followings:

**Query # 21**

Discussion lines 612-618, you have only present other results compared to your results but you did not discuss the possible explanations for the seasonal variation of Kala-azar observed in your study.

**Answer**

Agreed and added possible explanations for the seasonal variation of kala-azar from line no-678-688 at page no-41.

We know that two time (1^st^ round; March-May and 2^nd^ round; July-September) indoor residual spraying is being done every year in the endemic areas of kala-azar in Bihar state under kala-azar control programme of the country to control sand fly population. This is done in these months on the basis of our previous knowledge (earlier literatures) that pre and post monsoon season (February to August) sand fly resurgence is much high as compared to other seasons. Sand flies are found in abundance in endemic areas in rainy season due to favorable humidity and temperature, which include highest number of gravid sand flies. On the other hand, more active kala-azar cases are also found during January, March, July and August in the endemic areas. Therefore, there is chance of higher number of parasites in sand flies while taking a blood meal from an infected human host. Further, shorter incubation period (1-2 months) increased the disease transmission since February to August. Consequently, the infectivity of sand flies may be higher during these months.

**Query # 22**

Discussion part- Line 619- 630, you only mentioned the similarity and discrepancy of other studies compared to your result with regard to number of PKDL cases as double in Muzaffarpur and Samastipur district in the year 2013 when compared to 2011. However, you did not try to discuss the possible explanation for the high number of these two districts between 2011-2014.

**Answer**

Agreed and added possible explanations for the increased number of PKDL cases in Muzaffarpur and Samastipur districts from line no-691-692 at page no-41 and in line no-693-703 at page no-42.

**Query # 23**

Discussion, line 636-640, this is the first time that you give possible explanation for one of your results in the study namely gender differences of kala-azar proportion.

**Answer**

No response is needed.

**Query # 24**

Discussion part-line 695, If the  HMIS data was almost similar to the health facility sentinel surveillance data under the study of this manuscript, what is the need or advantage  for this sentinel surveillance unless there are  strong justifications need to be addressed by authors of this paper.

**Answer**

As suggested by the learned reviewer, justifications have been added from line no-830-850 at page no-48 and from line no-851-866 at page no-49.

Justifications are;

The major objectives of the study were proper accounting of VL, PKDL and co-infection morbidity pattern, mortality pattern, diagnosis, case management, hotspot identification and monitoring the impact of elimination interventions. Capturing complete and accurate patient level data in the existing surveillance system is always challenging. Generally treatment of unresponsive, relapse and re-infection cases were not managed at the existing health facilities under the program. Such cases mostly refer to higher health facilities (level-III facilities, which include medical colleges, kala-azar research institutions viz RMRIMS, KMRC, Balaji Utthan Sansthan, etc in the state). Our study managed 13.9% unresponsive; relapse and re-infection cases of VL at these sentinel sites. Study further captured 53 confirmed cases of PKDL with the help of slit-skin smear /skin-biopsy. The majority (98%) of patients with suspected VL; PKDL & Co-infections were managed in the sentinel sites itself. However, the detailed results are presented in “Results Section” and required justifications added in “Discussion Section”from line no-808-828 at page no-45 to line no-828-851 at page no-46.These data may be vital and missing in existing kala-azar surveillance system. Thus present study showed significant improvements in kala-azar, PKDL and co-infection cases management at the sites as compared to existing public health facilities. At the onset of the sentinel site, majority of suspected kala-azar cases were diagnosed and treated at the facility itself without further referral to elsewhere. Utilization of laboratory services were much improved at the sentinel sites. The use of diagnostic testing may improve patient care and reduce the uses of unnecessary anti KA drugs. Study further identified the hot spots of VL. These villages are crucial for the program manager and policy makers and focused intervention need to be initiated on urgent basis to meet the target of kala-azar elimination. The possible explanations may be the continued transmission of the disease due to favorable conditions to sand fly in these pockets and lacking of various program interventions. Data coming out from this study may also be vital for informing routine program decision making. It may further be useful in monitoring commodity stocks and demands. Program Managers may use results for addressing a variety of local needs on various aspects. This method may also be utilized in evaluating program effectiveness. The lessons learned from this study may benefit other initiatives aimed to improving kala-azar case management in other health care facilities. It may be helpful for changing the practices of health care workers in Bihar, India. Such systems may be utilized for deciding operational strategies to locate foci of increased KA, PKDL, and co-infection transmission. This system provided the basis that how fewer additional resources may make the system more sensitive/effective. It may also provide a model for improving laboratory services and KA, PKDL and co-infection case management in other health facilities in Bihar without further referral. Finally, it may possibly be used as a cost-effective approach for capturing real-time kala-azar data in national kala-azar elimination programme.

**Query # 25**

Conclusion- line 731, you advocated for the Health Facility-Based Sentinel Sites Surveillance System, my question how would such system will be sustained in the context of India, would you please elaborate on that at the text where you think is relevant.

**Answer**

Detailed Justifications for need or advantage of this study have been presented under “Query # 24”. The lessons learned from this study may improve kala-azar case management in other health facilities with fewer resources. This system provided the basis that how fewer additional resources may make the system more sensitive/effective. The drug and diagnostic are already available in most of the existing facilities. Laboratory personnel are also available in the health facilities. Most of the health system workers were utilized for this system. Additionally, only one site supervisor was recruited per district to monitor the activities carried out in the sentinel site. However, major concern was availability of pathologists in the existing health facilities. Mainly, specific training/re-training/orientation was provided to health facility workers under this system. This may be addressed by assigning responsibility and building strong coordination amongst existing health system workers, which may be assign to any suitable official available to health system. Frequent Training of laboratory personnel and clinician was needed to be conducted to make them efficient in discharging their assign duties. Training/re-training/orientation may change the practices of health care workers in Bihar, India. It also adds the dimension of collection and analysis of individual patient-related information. The magnitude of exiting health facilities problem can be assessed and an effective strategy may be evolved with the help of finding of this study. Therefore, sentinel site surveillance system may be sustained in the context of India and it would be relevant too in the light of findings of this study.

As suggested by the learned reviewer, elaborations have been added from line no-924-940 at page no-52.

Reviewer #3:

The comments given in the preceding should be attended to before the manuscript is accepted for publication.

**Answer**

Every comment given in the preceding has already been attended over the manuscript.
