# Supplementary material for: Improved kala-azar case management through implementation of health facility-based sentinel sites surveillance system in Bihar, India
Source: PLoS Negl Trop Dis. 2021 Aug 24;15(8):e0009598. doi: 10.1371/journal.pntd.0009598 (PMC8384155; doi:10.1371/journal.pntd.0009598)
Supplement: Attachment — Submitted filename: Reply to the reviewers comments R3.docx [file pntd.0009598.s003.docx]

**REPLY TO REVIEWER’S COMMENTS**

Reviewer #2: (No Response)

**Answer:** Not Applicable

Reviewer #3: The authors have taken the suggestions and improved the manuscript.

The manuscript can now be accepted for publication.

**Answer:** The authors would like to thanks the reviewer. No response is required.

Reviewer #2: (No Response)

**Answer:** Not Applicable

Reviewer #3: Yes, the presentation of results complies with above points.

**Answer:** The authors would like to thanks the reviewer. No response is required.

Reviewer #2: (No Response)

**Answer:** Not Applicable

Reviewer #3: Yes the conclusions are self explanatory.

**Answer:** The authors would like to thanks the reviewer. No response is required.

Reviewer #2: (No Response)

**Answer:** Not Applicable

Reviewer #3: Accept

**Answer:** The authors would like to thanks the reviewer. No response is required.

Reviewer #2: The authors have responded appropriately to the comments.

**Answer:** The authors would like to thanks the reviewer. No response is required.

Reviewer #3: The revised manuscript has improved.

**Answer:** The authors would like to thanks the reviewer. No response is required.
